# DNA methylation analysis identifies key transcription factors involved in mesenchymal stem cell osteogenic differentiation

**DOI:** 10.1186/s40659-023-00417-6

**Published:** 2023-03-08

**Authors:** Rodolfo Gómez, Matt J. Barter, Ana Alonso-Pérez, Andrew J. Skelton, Carole Proctor, Gabriel Herrero-Beaumont, David A. Young

**Affiliations:** 1 Musculoskeletal Pathology Group, Institute IDIS, Santiago University Clinical Hospital, Laboratorio 18, Edificio B, Planta -2, 15706 Santiago de Compostela, Spain; 2 Skeletal Research Group, Biosciences Institute, Newcastle University, Newcastle-upon-Tyne, NE1 3BZ UK; 3 Bioinformatics Support Unit, Faculty of Medical Sciences, Newcastle University, Newcastle-upon-Tyne, NE2 4HH UK; 4 Campus for Ageing and Vitality, Newcastle University, Newcastle-Upon-Tyne, NE2 4HH UK; 5 Bone and Joint Research Unit, IIS-Fundación Jiménez Díaz, UAM, 28040 Madrid, Avda Reyes Católicos Spain

**Keywords:** Osteoblastogenesis, Stem cell differentiation, Methylation, Bone adiposity

## Abstract

**Background:**

Knowledge about regulating transcription factors (TFs) for osteoblastogenesis from mesenchymal stem cells (MSCs) is limited. Therefore, we investigated the relationship between genomic regions subject to DNA-methylation changes during osteoblastogenesis and the TFs known to directly interact with these regulatory regions.

**Results:**

The genome-wide DNA-methylation signature of MSCs differentiated to osteoblasts and adipocytes was determined using the Illumina HumanMethylation450 BeadChip array. During adipogenesis no CpGs passed our test for significant methylation changes. Oppositely, during osteoblastogenesis we identified 2462 differently significantly methylated CpGs (adj. p < 0.05). These resided outside of CpGs islands and were significantly enriched in enhancer regions. We confirmed the correlation between DNA-methylation and gene expression. Accordingly, we developed a bioinformatic tool to analyse differentially methylated regions and the TFs interacting with them. By overlaying our osteoblastogenesis differentially methylated regions with ENCODE TF ChIP-seq data we obtained a set of candidate TFs associated to DNA-methylation changes. Among them, ZEB1 TF was highly related with DNA-methylation. Using RNA interference, we confirmed that ZEB1, and ZEB2, played a key role in adipogenesis and osteoblastogenesis processes. For clinical relevance, ZEB1 mRNA expression in human bone samples was evaluated. This expression positively correlated with weight, body mass index, and PPARγ expression.

**Conclusions:**

In this work we describe an osteoblastogenesis-associated DNA-methylation profile and, using these data, validate a novel computational tool to identify key TFs associated to age-related disease processes. By means of this tool we identified and confirmed ZEB TFs as mediators involved in the MSCs differentiation to osteoblasts and adipocytes, and obesity-related bone adiposity.

**Supplementary Information:**

The online version contains supplementary material available at 10.1186/s40659-023-00417-6.

## Introduction

Musculoskeletal disorders (MSD) are a set of pathologies that affect the locomotor system (bones, joints, peri-articular structures, and muscles). Research interest in these diseases has increased due to their elevated prevalence because of an ageing demographic resulting in a burgeoning economic cost to national health systems [1, 2]. In fact, 20% of Europeans undergo treatment or take supplements to help them cope with their MSD [3]. Besides ageing, the increase in the prevalence of major MSD conditions is also associated with an increase in obesity [4, 5] and a sedentary lifestyle [6, 7]. A common link amongst major MSDs are alterations to bone, such as osteoporosis and osteopenia, which have been associated with an increase in patient fragility [8] and in turn to a reduced life expectancy [9]. Bone alterations are also present in other MSDs, such as osteoarthritis where osteoporotic and sclerotic bone regions co-exist and both are linked to disease progression [10]. Likewise, in rheumatoid arthritis, as well as in other inflammatory arthropathies, there are local and systemic bone alterations [11].

Approximately 15% of the human skeleton is renewed every year [12]. This means that maintenance of an optimal bone mass depends on the precise balance between bone resorption and bone formation. When this balance is altered pathological conditions such as osteoporosis or osteopetrosis occur. Bone formation is carried out by osteoblasts, which share with adipocytes, their mesenchymal origin [12]. Therefore, the process of bone formation in turn depends on the balance between the differentiation of mesenchymal stem cells (MSCs) towards osteoblast or adipocyte cell fates [12]. It is considered that both processes, osteoblastogenesis and adipogenesis, are competing and reciprocal [12, 13]. In fact, bone marrow adiposity has been associated with bone loss in several MSDs as well as in other associated pathologies or conditions such as obesity and ageing [12, 14].

The commitment of the MSC towards osteoblast or adipocyte cell fate is controlled by certain transcription factors (TFs) such as runt related TF 2 (Runx2) and SP7 for osteoblastogenesis, and peroxisome proliferator activated receptor gamma (PPARγ) and C/EBPs [13] for adipogenesis. These TFs integrate the cell environment and the signalling of diverse pathways helping to initiate or block the differentiation process [13]. This can potentially have clinical implications for example, long-term pharmacological activation of PPARγ, which promotes adipogenesis and inhibits osteoblastogenesis, increases fracture rates among patients with diabetes [15]. Advances have been made investigating the role of TFs and their associated signalling networks in osteoblastogenesis and adipogenesis, however, despite the well-established transcriptional cascade associated with adipocyte differentiation [16] a better understanding of the TF network involved in osteoblast differentiation is required.

TF activity can be inhibited by DNA methylation through the blockade of their interaction with the DNA [17]. This inhibition has been associated with repression of gene expression, which supports the key role of DNA methylation in multiple processes, including development and tumorigenesis [17, 18]. DNA methylation is considered a heritable repressive epigenetic mark that consists of the covalent addition of a methyl group in the 5′ position of a cytosine in a CpG dinucleotide [18]. The abundance of these CpGs in the genome is not homogeneous, with more CpG-rich regions being present at TF-binding sites [17]. As a result DNA methylation has been proposed as a major regulator of TF activity [17].

There is limited information about the TFs that regulate the osteoblastogenesis process. Therefore, by means of a combined genome-wide methylation analysis and bioinformatic approach and using the link between DNA methylation and TF activity, we identified the TFs potentially affected by the changes in the DNA methylation during osteoblastogenesis. Our analysis suggested the DNA binding sites for the TF ZEB1/ZEB2 (Zinc Finger E-Box Binding Homeobox 1/2) were hyper-methylated due to the process of osteoblast differentiation. Functional data confirmed the role of these TFs on both osteoblastogenesis and adipogenesis processes, emphasising the relevance of DNA methylation on the activity of key TFs during cellular differentiation. Consistent with this, ZEB1 expression in human pathological bone samples revealed a potential link between this TF and metabolic-mediated bone alterations.

## Material and methods

### Reagents

IGF-1 was purchased from Peprotech (Rocky Hill, NJ, USA). DMEM, Foetal bovine serum, β-Glycerol Phosphate, dexamethasone, ascorbic acid 2-phosphate, insulin, 3-Isobutyl-1-methylxanthine, indomethacin, rosiglitazone, Cetylpyridinium, Oil Red, Alizarin Red were purchased from Sigma-Aldrich (St. Louise, MO, USA). Other products were also purchased from Sigma-Aldrich unless otherwise indicated.

### Cell culture

MSCs were purchased from Lonza (BAS, Switzerland). All the cells came from women donors, MSCs were cultured, characterized, and their trilineage potential was determined as previously described [19, 20]. The procedure for their culture and differentiation to osteoblast or adipocytes was performed as we previously described [21].

### Cytological staining

Cells undergoing osteoblast differentiation were fixed in 70% cold ethanol (5 min, − 20 ºC). After drying the wells, to reveal calcium-rich mineralisation deposits the cells were incubated at room temperature with a solution of Alizarin Red (40 mM, pH 4.2) for 20–30 min. Prior to acquiring the images, the cells were gently washed with distilled water to avoid unspecific staining. For quantitation, the staining was eluted with 10% (w/v) Cetylpyridinium solubilized in 10 mM sodium phosphate buffer (pH 7.0), and the absorbance measured at 570 nm.

Cells undergoing adipogenesis were fixed with formalin for 1 h. After washing the wells with distilled water and 60% isopropanol the wells were dried. To reveal the presence of lipid droplets the cells were stained with a 21% (w/v) solution of Oil Red O for 10 min. Prior to acquiring the images, the wells were gently washed with distilled water to avoid unspecific staining. Staining images were quantified using the image analysis software ImageJ.

### Bone samples

Bone samples from patients were obtained after total knee/hip replacement surgery for osteoarthritic and osteoporotic conditions. Healthy bone was from cadavers. Both healthy and pathological bone samples were comparable in terms of age and sex. The Ethics Committee for Research at Santiago-Lugo Area approved the protocol. Informed consent was obtained from all patients or patients’ families. Clinical data regarding weight and height was obtained from the clinical records, where available. BMI was calculated as weight (kg)/height (m)^2^.

To isolate bone RNA, bone explants were obtained assuring only trabecular bone was processed, thus without fat, cartilage, nor other fibroblastic or stromal tissues. Bone was repeatedly washed with Phosphate Buffered Saline until clean, frozen to − 80 ºC, and pulverised using a CellCrusher (Cellcrusher, Co. Cork, Ireland) following the manufacturer instructions. Per each 500 µl of bone powder, 1 ml of TriReagent was added to perform RNA extraction.

### RNA extraction and real-time reverse transcription PCR

Cell cultures were disrupted, and RNA extraction was performed using Qiagen RNeasy mini kit (Qiagen, Crawley, UK) following manufacturer instructions. Alternatively, when the experiments were performed in 96-well plates cell cultures were disrupted in Ambion Cells-to-cDNA II Cell Lysis buffer (Life Technologies, Carlsbad, CA, USA). Total RNA was then extracted and converted to cDNA using M-MLV reverse transcriptase (Invitrogen, Waltham, MA, USA) and RT-PCR was performed using TaqMan® probes. Changes in gene expression levels were calculated as described previously [21].

### DNA methylation and RNA expression arrays

Global DNA methylation analysis was performed using the Illumina Infinium HumanMethylation450K BeadChip array using DNA from three donor samples for each condition analysed (undifferentiated MSCs, MSCs differentiated to adipocytes, and MSCs differentiated to osteoblasts). All the samples were derived from the same donor to reduce variability. DNA, isolated using DNeasy Blood & Tissue Kit (Qiagen GmbH, Hilden, Germany), was bisulphite converted using the EpiTect® 96 Bisulphite Kit (Qiagen GmbH, Hilden, Germany), and 200 ng of bisulfite converted DNA was analysed using the array by the service provider Edinburgh Clinical Research Facility. The raw data were extracted using GenomeStudio (Illumina, San Diego, CA, USA) which provides the methylation data as β values: β = M/(M + U), where M and U represent the fluorescent signal of the methylation and unmethylated probes respectively. β values range from 0 (no methylation) to 1 (100% methylation).

For gene expression, an Illumina HumanHT-12 v4 Expression BeadChip array was performed using 200 ng of RNA [with an RNA integrity score > 7 (Agilent bioanalyzer 2100)] for each sample. Three samples from different donors and for each condition (undifferentiated MSCs, and MSCs differentiated to osteoblasts) were analysed. The RNA samples were processed according to the manufacturer’s protocol and the array was performed by Central Biotechnology Services, Cardiff University. Gene expression data were analysed essentially as previously described [19]. All data are available on request.

### Data analysis

HumanMethylation450K BeadChip data were normalised and analysed using the R language and the Tost analysis pipeline [22]. Further analyses to identify differentially methylated CpGs during differentiation were performed essentially using our own scripts developed from the limma package [23]. Data were transformed from β values to the more statistically valid M-values [24]. To explore the relationship between the distribution of differentially methylated CpGs and TF-binding and thus activity during the differentiation process we created an R-script, Regulatory Element Interrogation Script (REINS), available as an R Markdown document (Additional file 1). This script allows the download, management and overlap of the information provided by the ENCODE database [25], essentially ChIP-seq information about the genome-wide binding sites for 161 TFs in 91 different cell types, and differently methylated CpGs from any source including HumanMethylation450K BeadChip arrays. The script normalizes as a percentage the number of CpGs associated to a TF with the total number of CpGs. This allows the comparison of the overlap among different TFs with both hypo- or hyper-methylated CpGs. This normalization, “TF Relevance” (TFR), was performed using the following equations:

$$TFR\,\,hypomethylation = \frac{{no \,of\,hypomethylated\,CpGs\,overlapping\,the\,TF}}{{Total\,no \,of\,hypomethylated\,CpGs}}\times100$$

$$TFR\,\,hypermethylation = \frac{{no \,of\,hypermethylated\,CpGs\,overlapping\,the\,TF}}{{Total\,no \,of\,hypermethylated\,CpGs}}\times{100}$$

Finally, for the same TF, an unbalance in its ratio between TFRs for the hypo- and hyper-methylation (RRT, Relative Relevance of a TF) was calculated.

$$RRT\left(Relative Relevance of a Transcription Factor\right)=\frac{TFR\, hypomethylation}{TFR \,hypermethylation}$$

### Computational modelling

A computational model of BMP2 signalling was constructed to evaluate ALPL induction in the context of ZEB TFs activity. The design and construction of this model is described in Additional file 2.

### RNA-mediated interference (ZEBs expression inhibition)

For siRNA transfection 50 nM siRNA was transfected into 50% confluent MSCs using Dharmafect™ 1 lipid reagent (Thermo Fisher, Waltham, MA, USA). MSC were plated at a density of 5000 cells/well in 96-well plates for 24 h then media were replaced with osteogenic or adipogenic medium. After day 7 of differentiation the expression of key marker genes of differentiation was evaluated by RT-PCR. Cell cultures were cultured for 14 or 21 days to evaluate lipid accumulation or mineral deposition, respectively. Dharmacon siRNA SMARTpools® (Thermo Fisher, Waltham, MA, USA) of 4 specific siRNA duplexes (total of 50 nM siRNA) were used to target ZEB1 and ZEB2 TFs. Depletion of gene-specific mRNA levels was calculated by comparison of expression levels with cells transfected with 50 nM siCONTROL (non-targeting siRNA 2, cat. 001210–02; Dharmacon, Lafayette, CO, USA).

### Statistical analysis

Data are expressed as mean ± standard error of the mean (SEM) for at least 3 independent experiments. Statistical differences were determined using a one-way analysis of variance or Kruskal–Wallis test, followed by a Bonferoni or Dunn’s post-hoc test, respectively, or Student’s t test or Mann–Whitney when appropriate. Contingency table statistical analysis was performed using Fisher exact tests. Correlations were assessed with Spearman tests. Statistical analysis was performed using Prism software (GraphPad Software Inc), p < 0.05 was considered significant. The statistical analysis of the array was performed using R software and Bioconductor R-packages [23].

## Results

### Phenotypical characterization of MSC differentiation to osteoblasts and adipocytes

We examined the DNA methylation profile associated with the differentiation of human MSC to osteoblasts and adipocytes.

**Fig. 1:**
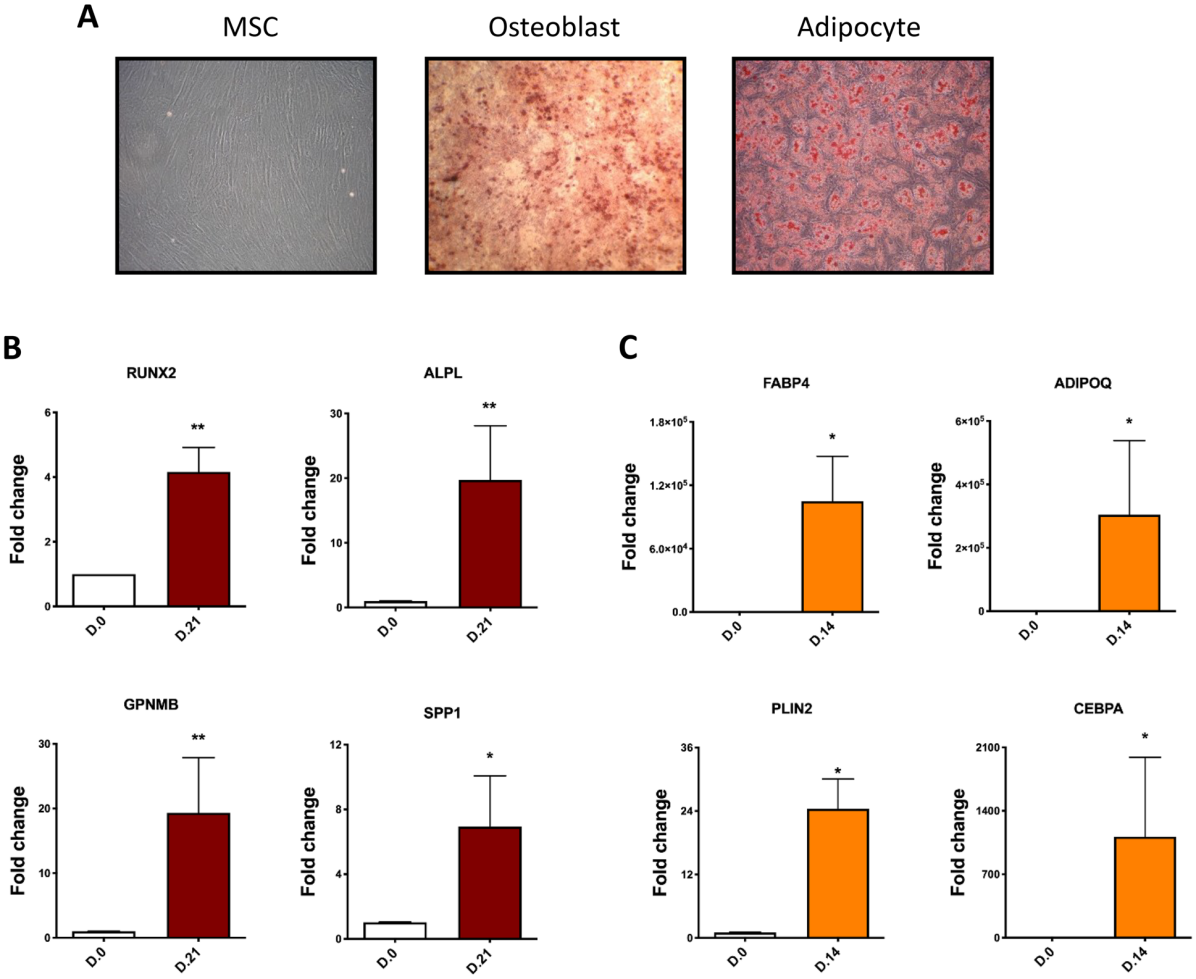
MSCs differentiation to osteoblasts and adipocytes. **A** Three donors MSCs differentiated to osteoblasts (21 days) and adipocytes (14 days) were stained with alizarin red and oil red, respectively. **B** Expression of osteoblast marker genes in MSCs differentiated to osteoblasts. **C** Expression of adipocyte marker genes in MSCs differentiated to osteoblasts. All the experiments were performed at least in 3 different donors. Data is expressed as mean ± SEM. * p < 0.05, **p < 0.01. All gene expression data were normalised to the reference gene, *GAPDH*, and expressed as fold relative to the control samples

MSCs were differentiated in adipogenic or osteoblastogenic media for 14 or 21 days, respectively. Differentiation was confirmed by cytological staining with alizarin red for osteoblast and oil red for adipocytes (Fig. 1A). To further confirm the differentiation well-characterised osteoblast and adipocyte differentiation marker genes were measured by RT-PCR with expression consistent with the expected differentiation status and staining (Fig. 1B and C).

### Global DNA methylation of MSC differentiation

**Fig. 2:**
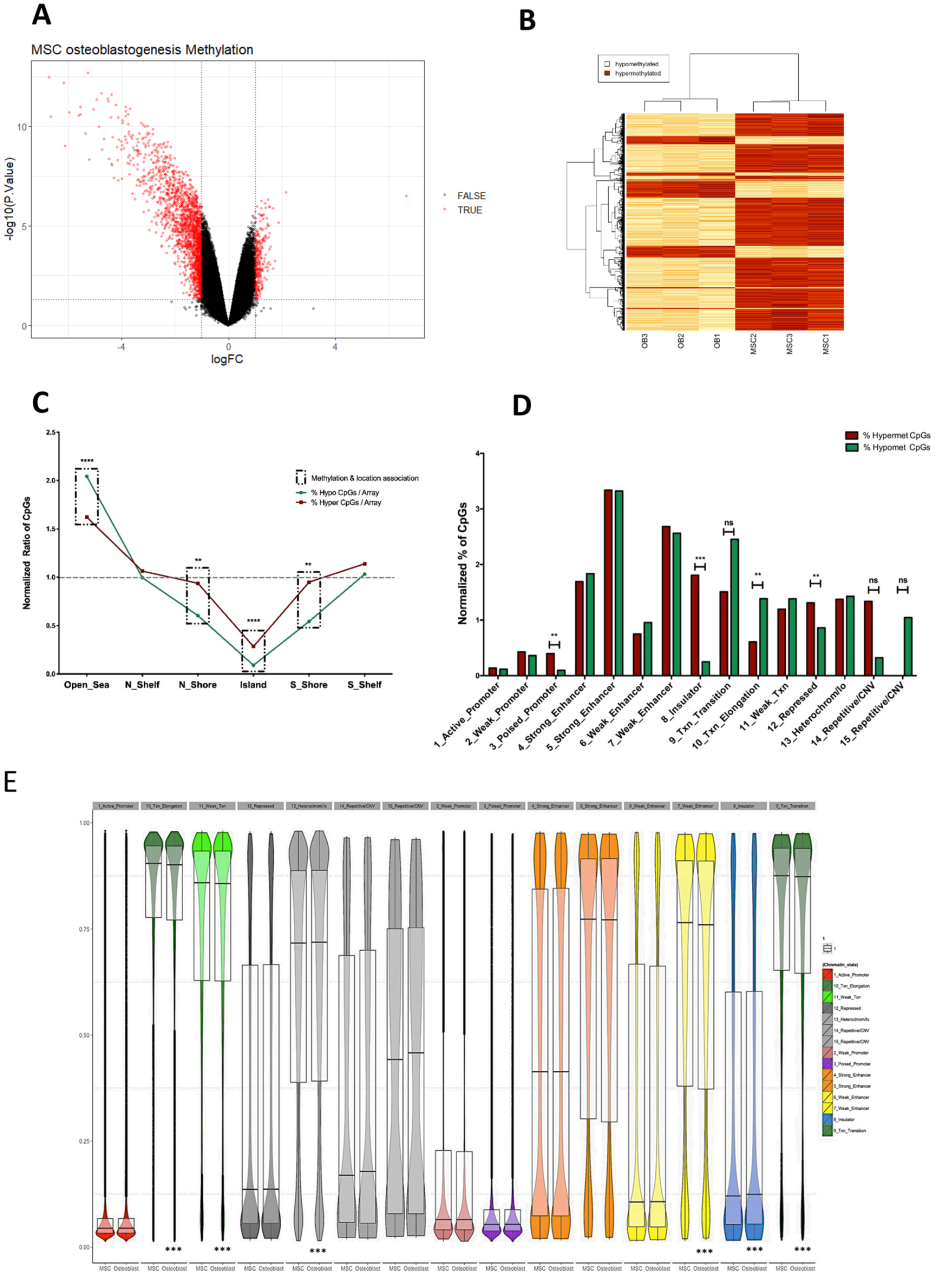
Analysis of the differently methylated CpGs. **A** Volcano plot representing the differentially methylated CpGs (M-values) associated with the differentiation of MSCs to osteoblasts. Significant (adjusted p ≤ 0.05) changes in CpG methylation (red). **B** Heatmap and clustering of the methylation values (M-values) of 2 462 CpGs differently methylated for MSCs differentiated to osteoblast. yellow: hypomethylated; red: hypermethylated. **C** Distribution of CpGs according to their location in relation to CpG islands. The number of CpGs were normalised according to the total CpGs in the array and the total amount of significant CpGs in each group (hypo- and hyper-methylated). Green line represents the percentage of hypo-methylated CpGs. Red line represents the percentage of hyper-methylated CpGs. Boxes identify a significant relationship between methylation and location. Broken grey line represents the expected distribution of the CpGs. ** p < 0.01, **** p < 0.0001. **D** Distribution of the differentially methylated CpGs according to the different chromatin states. The bars represent the percentage of significantly hypo- (green) and hyper- (red) differentially methylated CpGs normalised according to the number of CpGs present in each state (on the array). **E** Analysis of the methylation profile of all the CpGs studied in the array that overlap different chromatin states. Violin plots representing the 15 different chromatin states with methylation level represented from 0 to 1. For the statistical analysis a cut off of 0.1% in the difference of methylation was applied. The insulator state (p = 2.78E−2) and the heterochromatin state (p = 6.33E−37) were more methylated in OB than in MSC. The TXF elongation (p = 9.82E−30), TXF transition (p = 1.31E−09), and weak enhancer (p = 2.45E−06) states were less methylated in OB than in MSC. *** p < 0.001

DNA was extracted from MSC and differentiated cells and their global CpG methylation profile was assayed. For osteoblastogenesis, 2462 CpGs (1984 hypo-methylated and 478 hyper-methylated) showed a significant (adjusted p ≤ 0.05) change in their methylation (Fig. 2A) (Additional file 3). The overlapping genes of these CpGs (Additional file 3) included many in osteoblast metabolism such as *RUNX2*, *GPNMB*, *CTSK*, *WNT5A*, *COL1A1* and *PTH1R*. However, surprisingly, only significant changes in CpG methylation were observed for osteoblastogenic differentiation (Fig. 2B), which involves a limited role for DNA methylation in establishing and maintaining the adipogenic phenotype (Additional file 4) (Fig. 2B).

### Genomic topology of the differentially methylated CpGs during osteoblastogenesis

We examined the distribution of the 2462 differentially methylated CpGs within the genome and in relation to CpG island features. We found that osteoblastogenesis-dependent methylation remodelling was significantly associated with “open sea regions”. In contrast, methylation remodelling was less frequent than expected at CpG islands and their shores (Fig. 2C). Interestingly, only at “open sea regions” were the proportion of hypo-methylated CpGs higher than those hyper-methylated (Fig. 2C).

Chromatin profiling is a powerful tool to detect regulatory features within the underlying DNA [26]. Therefore, we overlapped the location of the osteoblastogenesis differentially hypo- or hyper-methylated CpGs with the location of the fifteen chromatin states used to segment the genome by ENCODE for the mesoderm cell-line GM12878 [26], normalising for CpG location frequency bias from the array. A significantly higher frequency of differentially methylated CpGs were located at enhancer, active transcription, and heterochromatin chromatin states (Additional file 6). CpGs defined to be located within insulator, poised promoter or polycomb-repressed states were more frequently hyper-methylated, while the reciprocal was evident at transcription elongation state where CpGs were more frequently hypo-methylated (Fig. 2D).

Next, we determined the methylation levels of all CpGs for each chromatin state in either the initial MSCs or following differentiation into osteoblasts. As predicted, promoter regions were very hypo-methylated while regions associated with transcription elongation or heterochromatin were hyper-methylated. When comparing CpG methylation levels for the two cell states, MSC and osteoblasts, data revealed that the methylation of the CpGs that overlapped with the insulator state and with the heterochromatin state were significantly higher in the osteoblasts than MSCs whilst those associated with chromatin states for transcription transition and elongation, and weak enhancer activity were lower (cut off 0.1% value; p < 0.001) (Fig. 2E).

### Correlation of DNA methylation and gene expression

**Fig. 3:**
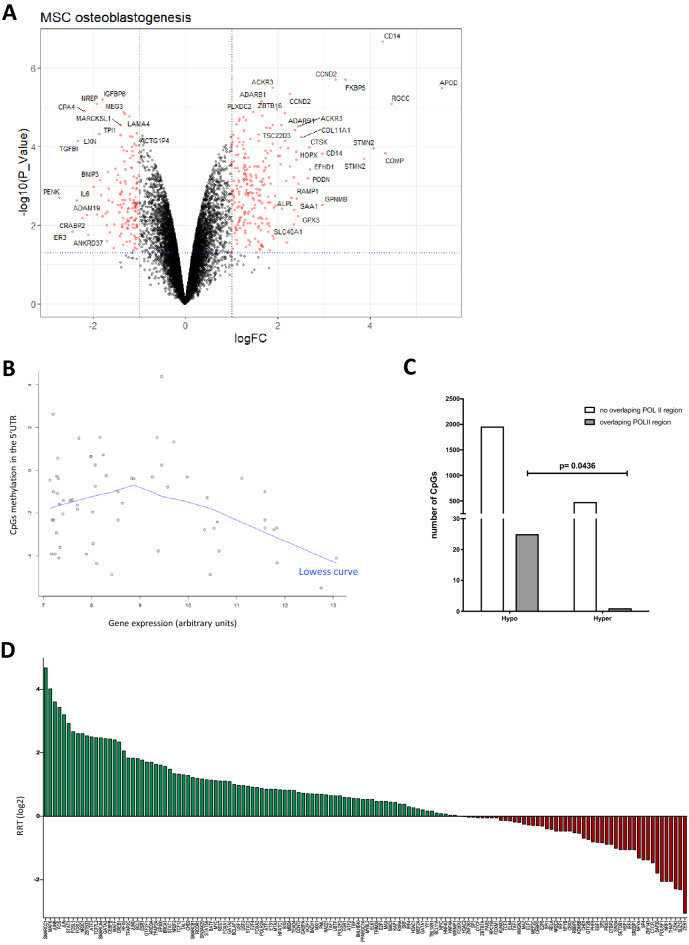
Relationship among methylation, gene expression and TFs. **A** Volcano plot representing the differentially expressed genes (adj. p ≤ 0.05, red) associated with the differentiation of MSCs to osteoblasts. **B** Lowess curve representing the correlation between the expression of genes in arbitrary units that had CpGs differently methylated in their 5’UTR and the methylation of these CpGs (M values). **C** Association of Hypo- and Hyper methylated CpGs to the chromatin interactions mediated by the RNA polymerase II (POL II). Data from chromatin Interaction Analysis by Paired-End Tag Sequencing (ChIA-PET) was obtained from ENCODE repository. Bars represent the number of CpGs that overlap (or not) to the POL II-mediated interactions. The methylation of CpGs overlapping or not POL II regions is significantly different p = 0.0436 (Fisher’s exact test). **D** Output of the REINS algorithm that overlaps hypo- of hypermethylated CpGs and TFs to suggest potential activity. Bars represent the metric RRT (Relative Relevance of a TF). RRT is the ratio of the percentage enrichment in hypomethylated CpGs vs the percentage enrichment in hypermethylated CpGs for a given TF. Bars represent the log2 of the RRT for all the TF studied

To correlate DNA methylation with gene expression, we performed whole genome transcriptome analysis of RNA from the donor MSCs and differentiated osteoblasts. This analysis identified 2315 genes/transcripts as significantly differentially expressed (adjusted p < 0.05) during the differentiation process (Fig. 3A) (Additional file 5). The analysis of this gene list using Ingenuity Pathway Analysis (IPA) identified that the major bone related pathways were significantly enriched (osteoblast differentiation, WNT pathway activation) (Table 1), (Additional file 7). Interestingly, there was also a significant enrichment in genes associated with adipogenesis inhibition (Table 1), supporting the fact that osteoblastogenesis and adipogenesis are opposing cell fates.

**Table 1.** Enrichment of the major bone related pathways

Pathway	p value
Cell differentiation	1.11E−26
Increased osteoblasts differentiation	9.80E−11
Increase differentiation of bone	3.94E−13
Increased alkaline phosphatase	2.17E−05
WNT pathway activation	6.46E−04
Inhibition of MMPs	1.41E−03
Inhibition of accumulation of lipids	8.56E−12
Inhibition of synthesis of lipid	1.07E−09

Upstream regulator	p value
TGFB1 inhibited	3.73E−26
ERBB2 inhibited	3.15E−23

Next, we explored whether gene expression and DNA methylation levels correlated in both MSCs and differentiated osteoblast. Using the methylation level of the significantly differentially methylated CpGs we observed a significant negative correlation only in osteoblasts (MSCs: r = − 0.0242, p = 0.6055 vs Osteoblasts: r = -− 0.377, p = 0.0031). Furthermore, when focussing on the methylation level of significantly differentially methylated CpGs that occur in 5’UTR regions, the negative correlation with gene expression improved for osteoblasts (r =  − 0.2500; p = 0.0499), in line with data for other cell-types [27]. This correlation further improved when analysing genes above a robust expression level threshold (> 1/3 of the maximum expression) (r = − 0.6289; p = 0.0013) (Fig. 3B).

### Osteoblastogenesis DNA methylation changes and POL II-chromatin interaction loci

DNA methylation changes do not correlate with the gene expression and biochemical changes that occur during adipogenesis. Similarly, though DNA methylation did associate with osteoblastogenesis the correlation with gene expression was low, except for the described 5’UTR gene region CpGs and genes with a robust expression level. Using Chromatin Interaction Analysis by Paired-End Tag Sequencing (ChIA-PET), Li et al. [28] defined regions of the genome, essentially enhancers, topologically-associated with RNA polymerase II (RNAPII). Following the same approach, we determined, using ENCODE data for the cell-line MCF7, if the genomic loci of our significantly differentially methylated CpGs during osteoblastogenesis were RNAPII bound regions -indicative of active transcription. We observed that there was a significantly different (p = 0.0436) association of hypo- versus hyper-methylated CpGs with RNAPII regions, with a greater overlap for hypo-methylated CpGs. (Fig. 3C)—implying that hypo-methylation of CpGs during osteoblastogenesis correlates with active gene expression.

### Identifying a link between DNA methylation and TF-binding sites

We investigated whether our DNA methylation signature could identify key TFs that drive or elicit MSC differentiation towards osteoblasts. To do this we designed an R script named REINS that overlaps and normalizes changes in DNA methylation with the TF-binding sites determined by ChIP-seq. For our analysis we used ChIP-seq data derived from the ENCODE project (161 TFs studied across 91 different cell-types). To validate the script we used publicly available data of DNA methylation from different tissues and cells, namely; adipose tissue, muscle, pancreas, thymus, and human pluripotent stem cells (hPSCs) [29]. The polycomb repressive complex 2 (PRC2) related TFs (EZH2, SUZ12, CTBP2) have been tightly associated with the epigenetic repression of stem cell genes during cell differentiation [30]. Thus, the identification of the differential activation state of these TFs [30, 31] on hPSCs (hypo-methylated > hyper-methylated) in comparison with other tissues (hyper-methylated > hypo-methylated) was considered a positive validation (Additional file 8A–C). Likewise, the prediction of the potential critical role of the transcription factor ZEB1 on the thymus (hypo-methylated > hyper-methylated) was also considered a positive validation (Additional file 8D). After the validation, we used REINS with the DNA methylation data of MSCs differentiated to osteoblasts. TFs were sorted according to their normalized ratio of enrichment for binding sites for hypo-/hyper-differentially methylated CpGs, with values > 0 more enriched for de-methylation and < 0 more enriched for methylation (Fig. 3D). The top significant (p < 0.001) TFs enriched, whose binding sites were relatively more hypo-methylated, included the bone anabolism related TFs SMARCC1, MAFK, JUNB, FOS, JUN and STAT3 [32]. Factors that promote osteoblast differentiation are frequently inhibitors of adipocyte differentiation, and vice versa [13]. Accordingly, we noted that a significant proportion of the top significant (p < 0.001) TFs whose binding sites were hyper-methylated were associated with the promotion of adipogenesis (EZH2, ZEB1, NFY, ZNF143, SIN3A, SREBP1, SUZ12, SAP30 and CTBP2) [33–39].

### Expression of ZEB TFs during MSC differentiation to osteoblasts

**Fig. 4:**
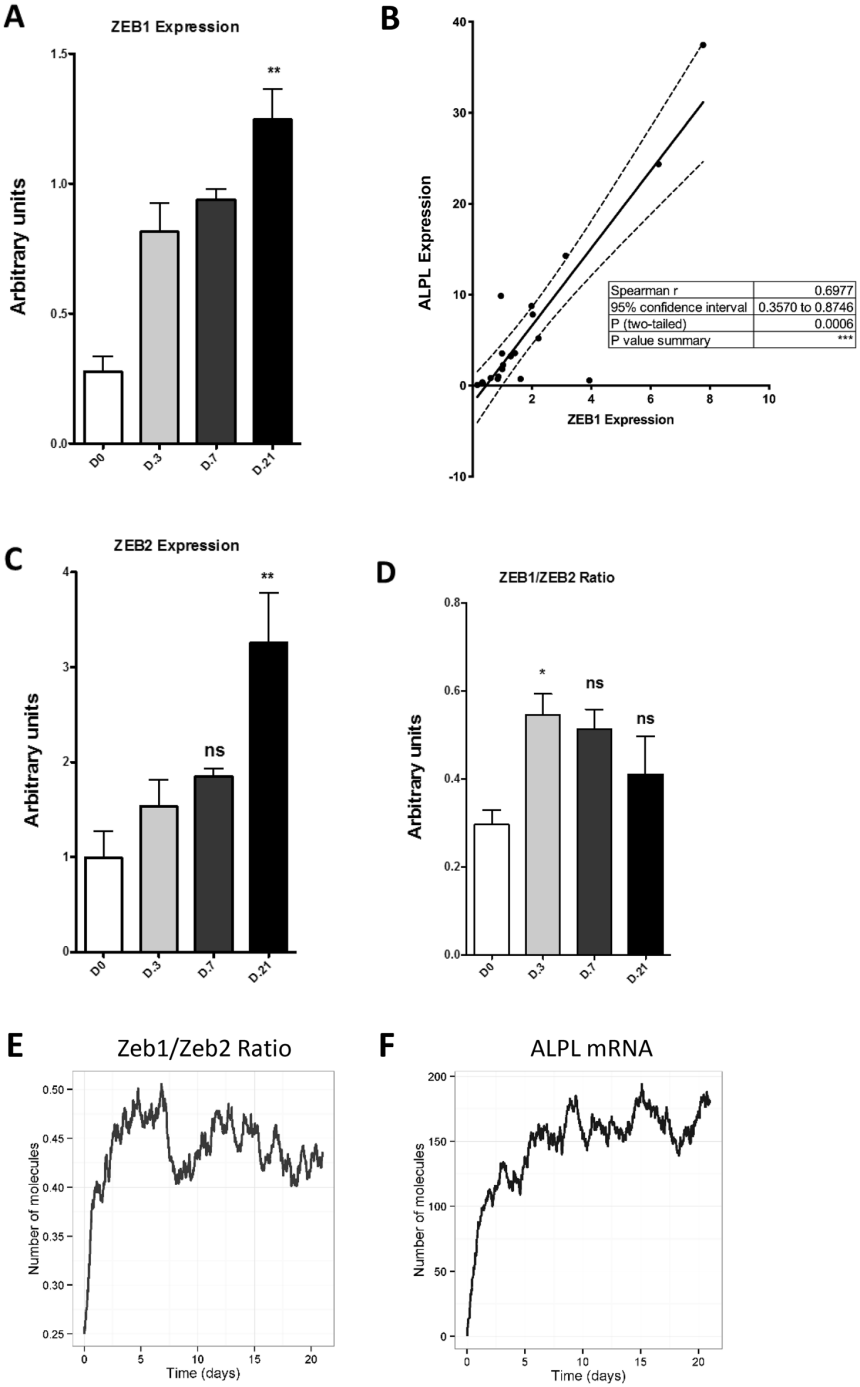
*ZEB1* and *ZEB2* expression during MSC differentiation to osteoblasts. **A** *ZEB1* gene expression represented in arbitrary units along the differentiation procedure. **B** Correlation of the gene expression of *ALPL* and *ZEB1* along the differentiation. Spearman r = 0.6977. p = 0.0006. **C** *ZEB2* gene expression represented in arbitrary units along the differentiation. **D** Ratio of induction of *ZEB1* and *ZEB2*. All the experiments were performed in at least 5 different donors. Data are expressed as means ± SEM. * p < 0.05, ** p < 0.01. **E** Output from one simulation of the computational modelling. The panel represents the ratio of the mRNA expression of *ZEB1*/*ZEB2* along 21 (virtual) days of differentiation. **F** Output from one simulation of the computational modelling. The panel shows the increased *ALPL* mRNA along 21 (virtual) days of differentiation

To validate the data obtained with REINS, we picked the TF most associated to DNA hyper-methylation during osteoblastogenesis, ZEB1 (log_2_RRT = -− 3.053). Thus, we evaluated the expression of *ZEB1* during our in vitro differentiation model. Counterintuitively, based on the methylation data, *ZEB1* mRNA expression increased during osteoblastogenesis (Fig. 4A), which was also positively correlated with the induction of *ALPL* expression, a differentiation marker (Fig. 4B). Interestingly, ZEB1 consensus binding sites can also be occupied by a related TF with repressor activity, ZEB2. *ZEB2* mRNA (ΔCt value) expression (data not shown) was relatively higher than *ZEB1* in MSC but showed a similar increase during osteoblastogenesis (Fig. 4C). However, early in the osteoblastogenesis the kinetics of *ZEB1* and *ZEB2* induction differed, as evidenced by the significantly different induction ratio ZEB1/ZEB2 at day 3 (Fig. 4D). To examine how the induction of both ZEB factors, and their different induction-kinetics, could be involved in osteoblastogenesis we constructed a computational model, which included known functions of ZEB1/ZEB2 in bone morphogenic protein (BMP) signalling [40] (Additional file 9). Since we only wanted to investigate the coexistence and interaction of both ZEB TFs in this model, we did not include other signalling pathways relevant for differentiation. Confirming the experimental data, in the model *ALPL* expression was induced during differentiation despite the presence of the repressor activity of ZEB2 (Fig. 4E, F), mainly due to the change in the induction kinetics of ZEB1 and ZEB2.

### Role of ZEBs TFs on MSC differentiation

**Fig. 5:**
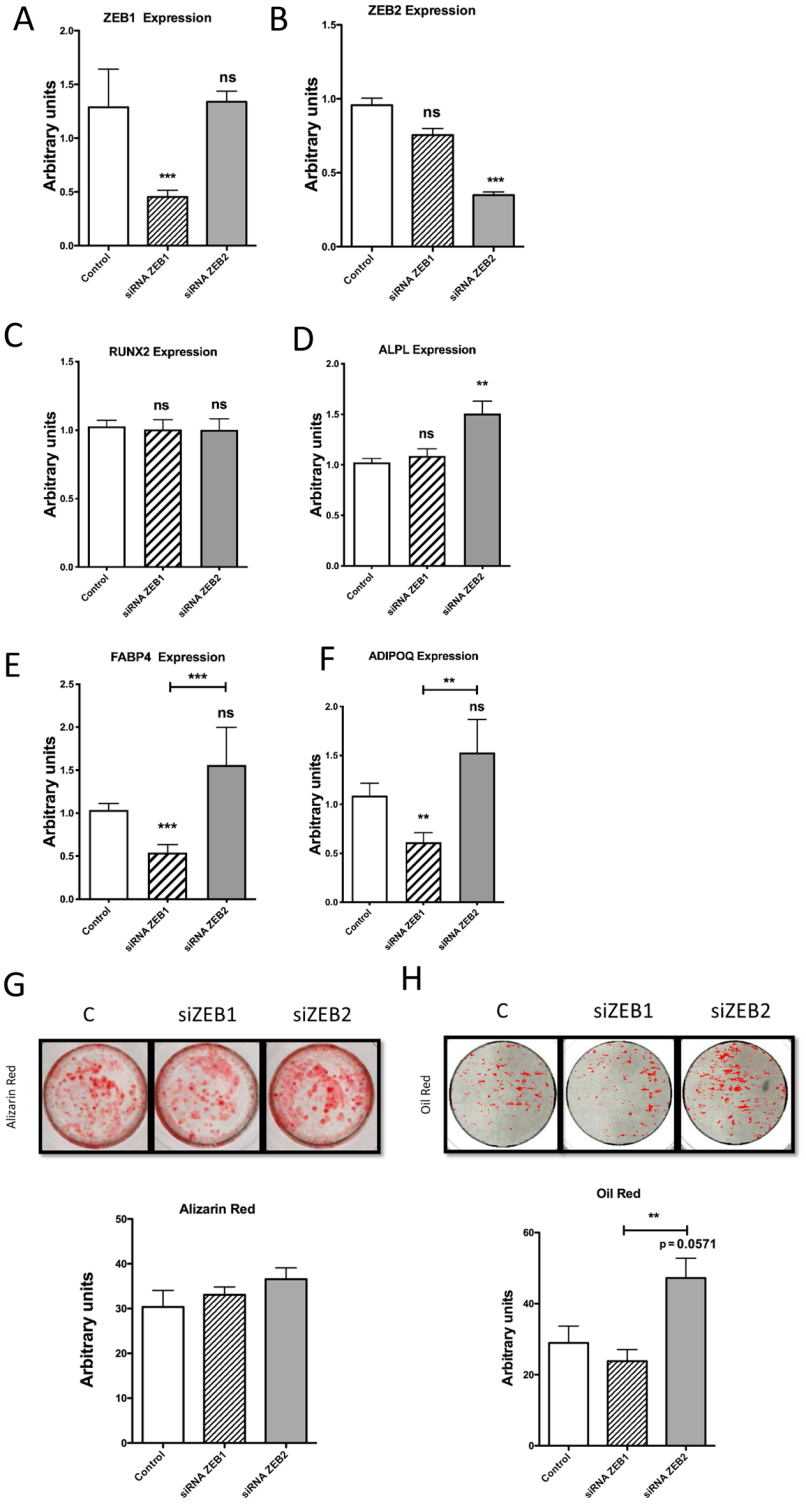
Expression of osteoblastogenesis and adipogenesis differentiation marker genes. Cells were treated with siRNA to deplete *ZEB1* and *ZEB2* mRNA expression, or Control non-targeting siRNA. **A** *ZEB1* and **B** *ZEB2* gene expression in control MSCs and MSCs treated with siRNA to knock-down *ZEB1* or *ZEB2* mRNA expression. All experiments were performed in at least in 4 donors. Data are expressed as mean ± SEM in arbitrary units. **C** *RUNX2* and **D** *ALPL* gene expression in 7 day differentiated MSCs to osteoblast. **E** *FABP4* and **F** Adiponectin gene expression in 7 day differentiated MSCs to adipocytes. All the experiments were performed with at least 4 donors. **G** and **H** Staining of calcium and lipid deposits in MSCs differentiated to osteoblasts and adipocytes. **G** Alizarin red staining of calcium deposits in MSCs differentiated to osteoblasts for 21 days. Cells were treated or not with siRNA to deplete *ZEB1* and *ZEB2* gene expression. Lower panel: alizarin red staining quantification expressed in arbitrary units. **H** Oil red staining of lipid deposits in MSCs differentiated to adipocytes for 14 days. Cells were treated or not with siRNA to deplete *ZEB1* and *ZEB2* mRNA expression Lower panel: oil red staining quantification expressed in arbitrary units. All the experiments were performed in at least 4 donors. Data are expressed as mean ± SEM in arbitrary units. ** p < 0.01, *** p < 0.001

Since ZEB1 is involved in both adipose and bone metabolism and the information on the function of ZEB2 is limited we studied the contribution of both TFs to the differentiation of MSC to adipocytes and osteoblasts. We depleted MSC of either *ZEB1* or *ZEB2* by siRNA prior to their differentiation to osteoblasts or adipocytes (Fig. 5A, B). After 7 days of differentiation the depletion of *ZEB1* expression did not alter the expression of osteoblast differentiation markers genes but did significantly reduce the expression of adipogenic markers (Fig. 5C–F). *ZEB2* depletion enhanced *ALPL* expression (Fig. 5C) but not *RUNX2* (Fig. 5D) and though adipogenic marker genes were increased this was not significant (Fig. 5E, F) unless compared to their expression level following *ZEB1* loss.

Although the transient depletion of either *ZEB1* or *ZEB2* did not significantly alter the mineralization in fully differentiated (day 21) osteoblasts (Fig. 5G), significant differences were observed in the lipid accumulation between fully differentiated adipocytes with an inhibited expression of *ZEB2* compared with *ZEB1* (Fig. 5H). These data again pointed that the alteration of *ZEB1*/*ZEB2* relative levels at the start of differentiation affected the outcome of the adipogenic process.

### Bone ZEB1 expression positively correlates with body weight and BMI

**Fig. 6:**
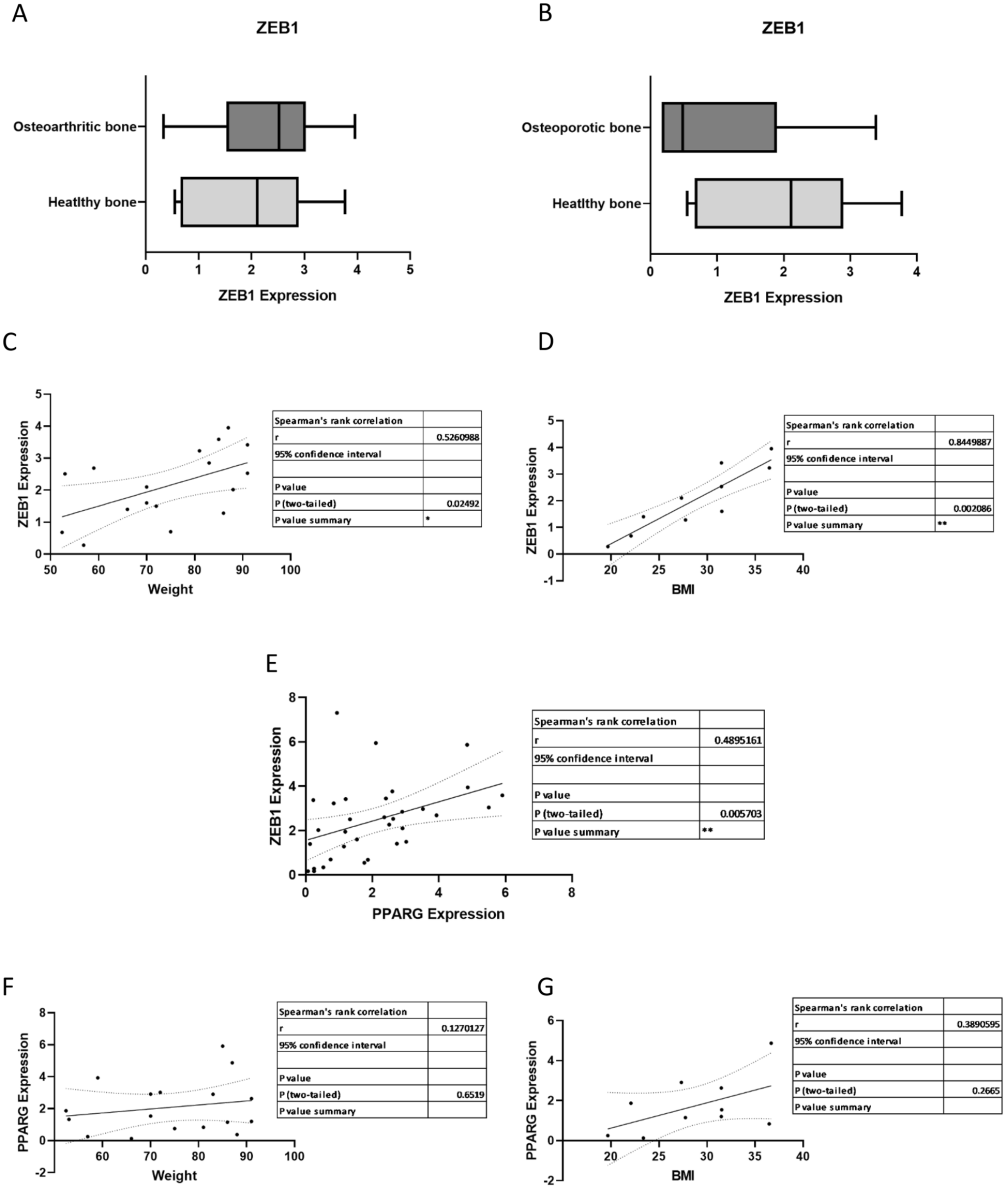
*ZEB1* expression in bone correlates with donor weight and BMI, but not disease status. **A** *ZEB1* expression in bone from osteoarthritic patients and patients without bone-related pathologies Healthy bone n = 6, Osteoarthritic bone n = 7, Osteoporotic bone n = 6. Data are expressed as mean ± SEM in arbitrary units. **B** *ZEB1* expression in bone from osteoporotic patients and patients without bone-related pathologies. Data are expressed as mean ± SEM in arbitrary units. **C** Correlation of *ZEB1* expression in bone vs donor weight (kg). **D** Correlation of *ZEB1* expression in bone vs BMI (weight (kg)/height (m)^2^). **E** Correlation of *ZEB1* expression in bone vs expression of *PPARG*, a major adipogenic inducer. **F** Correlation of *PPARG* expression in bone vs weight (kg). **G** Correlation of *PPARG* expression in bone vs BMI. * p < 0.05, ** p < 0.01

Regarding the observed implication of ZEB1 in adipogenic metabolism, we aimed to determine whether the expression of this TF was modulated in certain pathologies or physiological situations where bone integrity is affected. Neither osteoarthritic nor osteoporotic bone exhibited variations of *ZEB1* expression in comparison to healthy bone (Fig. 6A, B).

As previously described, ZEB1 is involved in obesity development. Considering that obesity is defined as increased fat accumulation, we used body weight to investigate whether bone *ZEB1* expression was linked to this pathology, identifying a significant correlation between the expression of this TF and body weight (Fig. 6C). Moreover, to discriminate among healthy, overweight, and obese patients we explored the same correlation using BMI instead of weight, which further improved the correlation coefficient (Fig. 6D).

Obesity has been associated with bone adiposity [12]. Likewise, bone adiposity has been linked to the activation of PPARγ [41]. Thus, we aimed to determine whether *PPARG* expression correlated with *ZEB1* expression in bone samples. Data obtained showed a positive correlation between both genes (Fig. 6E), however *PPARG* expression did not correlate with weight nor BMI (Fig. 6F, G), indicating a relevant role for ZEB1 in obesity-associated bone alterations.

## Discussion

Here we have described the methylation profile of MSCs undergoing osteoblastogenesis and shown that changes in DNA-methylation occur outside of CpG islands. Likewise, we have associated these methylation changes to established chromatin states and linked the changes in DNA-methylation and gene expression. We have also developed a software tool to investigate the relationship between TFs and DNA-methylation profiles, which we used to define a set of candidate TFs potentially linked to the activation or repression of osteoblastogenesis. Accordingly, we validated the role of one of these inactivated TFs, ZEB1, and its opposing counterpart, ZEB2, on the differentiation of MSCs. We observed that modulating the ratio of these opposing factors affected both osteo- and adipogenesis, experimentally validating our bioinformatic tool.

Bone alterations are a common link amongst many musculoskeletal pathologies and have been associated with increased patient fragility [8]. Further, bone-marrow adiposity increases with age in part because of an increase in MSC differentiation to adipocytes rather than osteoblasts [4, 42, 43]. Epigenomic state has been studied and no significant methylation changes were found in adipogenesis [44, 45], however, similar osteoblastogenic information is limited [12]. As a result, we determined DNA-methylation signature of both processes with an aim of identifying regulatory elements and TFs involved.

In this work we were able to determine the changes in the methylation profile associated with MSC osteoblastogenesis, which revealed that the methylation remodelling occurred outside of the CpG islands, consistent with previous reports describing methylation differences between tissues [46], and suggested that DNA methylation could contribute to, or reinforce, the differentiation process. Additionally, by overlaying chromatin profiling data [26], we observed that osteoblastogenic DNA methylation remodelling (both hypo- and hyper-methylation) occurred at enhancer chromatin states. Interestingly, an increase in methylation was evident in the CpGs of insulator, poised promoter, and polycomb-repressed states, with the opposite true for transcription elongation state. This not only added a new layer of evidence to the involvement of DNA methylation on the regulation of osteoblastogenesis but also described the insulators as important points of regulation. DNA-binding of the well-characterized insulator CCCTC-binding factor is highly sensitive to DNA methylation [47], which suggests that osteoblastogenesis-associated methylation changes could alter CCCTC-binding factor binding, and therefore alter 3D genome architecture and thus influence function [47–49].

Unexpectedly, we did not identify any significant changes in the DNA methylation profile during adipogenesis. Supporting this observation, Noer et al. [50] described that DNA methylation of adipogenic promoters did not reflect transcriptional status, nor potential for gene expression, in adipocytes differentiated from MSCs. Likewise, it was determined that DNA methylation remained stable during adipocyte differentiation, implying that DNA methylation may not be a determinant of the adipogenic differentiation process [45]. Conversely, crude pharmacological inhibition of DNA methylation (with 5-aza-2′-deoxycytidine) in 3T3-L1 preadipocytes inhibited adipogenesis but promoted osteoblastogenesis [51]. MSCs are clearly delicately balanced for their differentiation commitment with regards adipo-osteogenic differentiation of MSCs [12, 13]. Our data suggests that adipogenesis can be achieved without a substantial modification of the default methylation profile of MSCs and that this phenotype is somewhat plastic.

When MSC undergo osteogenic differentiation, the phenotype may be more stable due to its maintenance through a defined DNA methylation programme. Accordingly, during osteoblastogenesis we observed a negative correlation between DNA methylation and medium to highly expressed genes, and a significant link between DNA hypo-methylation and DNA regions related to active gene expression (RNAPOLII regions). Supporting this hypothesis we observed through our novel bioinformatic tool “REINS” that DNA hyper-methylation during osteoblastogenesis was enriched at binding sites of several pro-adipogenic transcriptional regulators [33–39, 52–54]—thus having the potential to inhibit their DNA-binding and therefore activity. Also enriched in osteoblastogenic hyper-methylated regions were binding sites for the transcriptional repressor SETDB1, a H3–K9 histone methyltransferase. SETDB1 binds to methylated DNA via a methyl-CpG-binding domain [55], thus enrichment of this factor is consistent with its described role as an inhibitor of adipogenesis [56]. Although REINS was validated because it identified the inactive epigenetic repression state of PRC2 TFs on hPSCs, the script is currently limited to the data of the 161 TFs present in the ChIP-seq data from the ENCODE project. As a result, the analysis is biased against TFs that regulate osteoblast metabolism and differentiation for which ChIP-seq data is lacking.

Among the TF potentially inactive or repressed during the osteoblastogenesis, we studied ZEB1, which had the largest enrichment in the ratio DNA hyper-methylated/ hypo-methylated predicted by REINS. ZEB1 is a zinc-finger protein involved in adipocyte differentiation in mouse, as well as in obesity development in humans [33, 57]. It is also involved in thymus and skeletal development [58, 59] with its deletion in mouse being related to craniofacial abnormalities (shortened jaw), limb defects, and fusion of ribs [59]. Consistent with these data, REINS also predicted the potential activation state of this TF on thymus and adipose tissues (Additional file 8D). However, the potential inactive state predicted for ZEB1 in osteoblastogenesis by the tool was contrary to its reported activities promoting BMP2-mediated *ALPL* expression [40], skeletal abnormalities presented in ZEB1-null mice [59], and our observed increase in *ZEB1* mRNA expression during osteoblastogenesis, which correlated with ALPL mRNA expression. In fact, ZEB1, along with orthologous ZEB2, are members of the ZEB family of transcription factors, and both can bind to E-box sequences CACCT(G). ZEB2 deficiency in humans has also been associated with craniofacial abnormalities, such as the excessive growth of the jaw (Mowat-Wilson syndrome) [60]. Interestingly, the functionality of ZEB proteins appear cellular-context dependent and, of relevance here, ZEB1 and ZEB2 have reported opposing activities (enhancer and repressor, respectively) upon activation of osteoblastic TGFβ or BMP signalling pathways [40, 61, 62]. Bone marrow has a higher level of ZEB2 expression compared to ZEB1 expression [63], which is consistent with their expression levels in our MSCs. Although both factors increased in mRNA expression during osteoblastogenesis, the ratio of these inductions (ZEB1 vs ZEB2) significantly increased at day 3 of differentiation. These data may suggest ZEB1 could be more active in early differentiation. At the late stages of differentiation, however, when ZEB2 would be predicted to dominate, we found a potential link of ZEB binding sites with hyper-methylation, which we speculate is to reduce the impact of ZEB2 acting repressively on osteoblastogenesis or to regulate the potential ZEB1-mediated adipogenic behaviour described previously [33, 57]. In line with this, our computational model revealed that the presence of the repressive ZEB2 to be compatible with the induction of *ALPL* along the differentiation process.

To examine this hypothesis, we depleted ZEB1 and ZEB2 in MSCs prior to inducing their differentiation to osteoblasts and adipocytes. Removal of ZEB2 enhanced expression of the osteoblast differentiation marker *ALPL*, again supporting its role as an osteoblastogenic repressor. Interestingly, *ZEB1* depletion did not affect osteoblast differentiation or mineralization suggesting that the role of ZEB1 during the process could be limited or only evident upon removal of the repressive ZEB2.

To the contrary, ZEB1 depletion in MSCs significantly reduced the expression of the adipogenic differentiation markers, consistent with its role as an adipogenic promoter [33]. ZEB1 depletion partially reduced lipid accumulation, though this did not reach significance possibly due to the transient nature of siRNA-mediated depletion after the 14 days of differentiation required for lipid generation. Meanwhile, *ZEB2* depletion increased adipogenic gene expression and oil staining when compared with the levels in *ZEB1* depleted cells, again suggesting that the modulation of the ratio of the expression of these TFs in the MSCs regulates the adipogenic process.

Regarding the essential role of ZEB1 in adipogenesis, we observed a positive correlation between bone *ZEB1* expression and body weight or BMI. These data are consistent with the described relation between ZEB1 and obesity [57] and evidence how this metabolic pathology not only affects fat tissue, but also bone. Underpinning these correlations, we have found that *ZEB1* also correlated with *PPARG* in bone samples. In agreement with this, it has been reported that *ZEB1* knockdown diminished *PPARG* expression in 3T3-L1 cells [33]. Since *PPARG* is a major adipocytic inducer, and its activation has been associated with bone adiposity and fragility [15], the positive correlation of ZEB1 with this TF could support the role for ZEB1 in bone adiposity. Interestingly, PPARG expression did not correlate with weight or BMI, which suggests that the obesity effects on bone adiposity might imply ZEB1 modulation rather than major adipogenesis inducer *PPARG*.

In conclusion, in this work we have characterised the DNA-methylation changes that occur during MSC-osteoblastogenesis. We propose that DNA methylation may be important for imprinting and maintaining the osteoblastic phenotype but not that of adipocytes. We present a script that could help to identify key TFs-associated with a given process through the analysis of DNA-methylation. Moreover, we have validated the algorithm on tissue-specific and osteoblastogenic DNA-methylation signatures, identifying ZEB TF ratios as modulators of differentiation of MSCs to osteoblasts and adipocytes, and ZEB1 as a critical TF in obesity-related bone adiposity.

## Supplementary Information

**Additional file 1: **REINS NCL 2020. Script, Regulatory Element Interrogation Script (REINS), available as an R Markdown document, to explore the relationship between the distribution of differentially methylated CpGs and TF-binding and thus activity during the differentiation process.

**Additional file 2: **Model design. A computational model of Bmp2 signalling was constructed to evaluate ALP induction in the context of ZEB TFs activity.

**Additional file 3: **CPG list and overlapping genes. List of differently methylated CPGs associated to osteoblast differentiation and their overlapping genes.

**Additional file 4: **CpG_OB_AP_MSC. Methylation status (M-values) of CPGs differently methylated across the MSC differentiation to osteoblast and adipocyte.

**Additional file 5: **RNA array. Differently expressed genes between stem cells differentiated (21 days) or not to osteoblast.

**Additional file 6: Figure S1.** Observed vs expected ratio of CpGs enrichment among the different chromatin states and their cumulative methylation profile in MSCs. A) The bars represent the ratio of the percentage of differently methylated CpGs vs the percentage of the total CpGs studied for each chromatin state. B) Cumulative plot of the methylation of the CpGs overlapping top enriched (Strong_ Enhancer, Weak_ Enhancer, and Txn_Transition) and depleted chromatin states (Active_promoter and Poised_ Promoter). Y axis: cumulative percentage of CpGs overlapping to each chromatin state. X axis: fold change methylation MSCs vs Osteoblasts.

**Additional file 7: Figure S2.** Output of ingenuity pathway analysis. Representation of the significant enrichment on ‘inhibition of MMPs’ as well as ‘WNT pathway activation’.

**Additional file 8: Figure S3.** Validation of REINS algorithm. Transcription factor relevance (TFR) metric for hypo (Ho)- and hypermethylated (Hr) CpGs associated to MSCs differentiation to osteoblasts (OB), to different tissues (muscle, adipose tissue, pancreas, thymus) and to human pluripotent stem cells (hPSC). A) TFR for the repressor TF CTBP2. B) TFR for the TF EZH2 a member of the polycomb repressive complex 2 (PRC2). C) TFR for the TF SUZ12 a member of the polycomb repressive complex 2 (PRC2). D) TFR for the TF ZEB1.

**Additional file 9: Figure S4.** Computational simulation of Zeb1 and Zeb2 interaction in the context of BMP2-induced target gene expression.

## Data Availability

The datasets generated during and/or analysed during the current study are not publicly available but are available from the corresponding author on reasonable request.
