# Supplementary material for: DNA methylation analysis identifies key transcription factors involved in mesenchymal stem cell osteogenic differentiation
Source: Biol Res. 2023 Mar 8;56:9. doi: 10.1186/s40659-023-00417-6 (PMC9996951; doi:10.1186/s40659-023-00417-6)
Supplement: Supplementary file 1 — Additional file 1: REINS NCL 2020. Script, Regulatory Element Interrogation Script (REINS), available as an R Markdown document, to explore the relationship between the distribution of differentially methylated CpGs and TF-binding and thus activity during the differentiation process. [file 40659_2023_417_MOESM1_ESM.html]

Regulatory Element Interrogation Script (REINS)


# Regulatory Element Interrogation Script (REINS)

#### Rodolfo Gómez

#### 11/3/2020

### 1. **Description**

This is an R script, presented as an R Markdown document, to explore the distribution of deferentially methylated CpGs in the context of transcription factor(TF)-binding regions (ChIP-seq determined) and thus infer their activity during in a giving process or condition. This script allows the download, management and overlap of the information provided by ENCODE database (Gerstein, M.B. et al. Nature. 2012) (ChIP-seq track about the genome-wide biding sites for 161 TFs in 91 different cell types) and differently methylated CpGs from any source, including HumanMethylation450K BeadChip arrays. Additionally, this script normalizes as percentage the number of CpGs associated to a TF with the total number of CpGs. This allows the comparison of the overlapping hypo- or hyper-methylated CpGs among different TFs and different processes.

### 2. **REINS requirements**

- #### Dependencies installation

  This is only required the first run of REINS.

```
if (!requireNamespace("BiocManager", quietly = TRUE))
  install.packages("BiocManager")
BiocManager::install()
library(BiocManager)
BiocManager::install("GenomicFeatures")
BiocManager::install("IRanges")
BiocManager::install(c("methylumi", "lumi"))
BiocManager::install("qvalue")
BiocManager::install("limma")
BiocManager::install("sva")
BiocManager::install("lumiHumanIDMapping")
BiocManager::install("FDb.InfiniumMethylation.hg19")
BiocManager::install("rtracklayer")
BiocManager::install("rlist")
```

- #### Libraries load

```
library(stringr)
library(sva)
library(lumi)
library(gplots)
library(annotate)
library(ggdendro)
library(limma)
library(lumiHumanIDMapping)
library(FDb.InfiniumMethylation.hg19)
library(rtracklayer)
library(GenomicFeatures)
library(GenomicRanges)
library(lumi)
library(rlist)
```

- #### CpGs genomic data download

  We download the data of the CpGs genomic ranges from illumina array.

```
InfiniumMethylation <- features(FDb.InfiniumMethylation.hg19)
  met <- metadata(FDb.InfiniumMethylation.hg19) ## need to fetch genome
          genome(InfiniumMethylation) <- met[which(met[,"name"]=="Genome"),"value"]
          InfiniumMethylation <- sort(InfiniumMethylation)
          show(InfiniumMethylation)
```

```
## GRanges object with 487173 ranges and 14 metadata columns:
##              seqnames            ranges strand | addressA_450 addressB_450
##                 <Rle>         <IRanges>  <Rle> |  <character>  <character>
##   cg01686861    chr16       60748-60749      + |     36644319     45624454
##   cg05558259    chr16       61085-61086      + |     65765435             
##   cg26978960    chr16       62460-62461      + |     28717484             
##   cg03792876    chr16       73243-73244      + |     42725455             
##   cg09699726    chr16       91602-91603      + |     34629364             
##          ...      ...               ...    ... .          ...          ...
##   cg13808036     chrY 24453658-24453659      - |     47649309             
##   cg26983430     chrY 24549675-24549676      - |     24631411     26605360
##   cg17939569     chrY 27009430-27009431      - |     73757458             
##   cg13365400     chrY 27210334-27210335      - |     61745505             
##   cg14273923     chrY 28555912-28555913      - |     16749405             
##              addressA_27 addressB_27  channel450   channel27   probeType
##              <character> <character> <character> <character> <character>
##   cg01686861        <NA>        <NA>         Red        <NA>          cg
##   cg05558259        <NA>        <NA>        Both        <NA>          cg
##   cg26978960        <NA>        <NA>        Both        <NA>          cg
##   cg03792876        <NA>        <NA>        Both        <NA>          cg
##   cg09699726        <NA>        <NA>        Both        <NA>          cg
##          ...         ...         ...         ...         ...         ...
##   cg13808036        <NA>        <NA>        Both        <NA>          cg
##   cg26983430        <NA>        <NA>         Red        <NA>          cg
##   cg17939569        <NA>        <NA>        Both        <NA>          cg
##   cg13365400        <NA>        <NA>        Both        <NA>          cg
##   cg14273923        <NA>        <NA>        Both        <NA>          cg
##                percentGC    platform
##              <character> <character>
##   cg01686861        0.76       HM450
##   cg05558259        0.56       HM450
##   cg26978960        0.66       HM450
##   cg03792876        0.64       HM450
##   cg09699726        0.68       HM450
##          ...         ...         ...
##   cg13808036        0.64       HM450
##   cg26983430        0.56       HM450
##   cg17939569        0.42       HM450
##   cg13365400        0.44       HM450
##   cg14273923        0.48       HM450
##                                                       sourceSeq  probeStart
##                                                     <character> <character>
##   cg01686861 CGCCCCCAGGCCGGCGCCGTGCGACTTTGCTCCTGCAACACACGCCCCCC       60700
##   cg05558259 CAGCTAGGGACATTGCAGGCTCCTCTTGCTCAAAGTGTAGTGGCAGCACG       61037
##   cg26978960 CGGCCCAGTAGAGCCCTAGGGGTGACGCCACTCCCACTCACTGTCGACTC       62412
##   cg03792876 ATGGAGGCTTGGGCGGGTCACCCCCAGTGCAGGCCAAGATGCAGGTTACG       73195
##   cg09699726 GCACTAGAGCCCCAGGGCCAGGGGAGACCCAGCATCCAAGGTGCACCACG       91554
##          ...                                                ...         ...
##   cg13808036 CGGCGCCCACCCACTGCTGCCAGCCATCCCGAATTGACAGCTGCAAGGAT    24453658
##   cg26983430 CGTACGCCTGAGGGCCAGGCGAACCTCAGGCTCTTTGTCCTACTAAAAAG    24549675
##   cg17939569 CGCCTAAATAAGAATAGGAGTAAAGGAGAGTATTACCTCCAAATCACCGG    27009430
##   cg13365400 CGTCACCTGGATGCTGGTTTAAGTGATATATGAAAATCCACCCTAAGGAC    27210334
##   cg14273923 TGGTATTGGTGAAGTCTACCACTCCAGCTCGTAGACTTCCATAATCGTCG    28555912
##                 probeEnd probeTarget probeExtension
##              <character> <character>    <character>
##   cg01686861       60749       60748        60749.0
##   cg05558259       61086       61085        61086.0
##   cg26978960       62461       62460        62461.0
##   cg03792876       73244       73243        73244.0
##   cg09699726       91603       91602        91603.0
##          ...         ...         ...            ...
##   cg13808036    24453707    24453658           <NA>
##   cg26983430    24549724    24549675           <NA>
##   cg17939569    27009479    27009430           <NA>
##   cg13365400    27210383    27210334           <NA>
##   cg14273923    28555961    28555912           <NA>
##   -------
##   seqinfo: 24 sequences from hg19 genome; no seqlengths
```

- #### TFs genomic data download

  We download the data of TFs genomic ranges from ENCODE database. The dowload of the data usually takes a lot of time.

```
          fdb<-makeFeatureDbFromUCSC(genome="hg19", 
              track="Txn Factor ChIP",
              tablename="wgEncodeRegTfbsClusteredV3")
```

```
## Download the wgEncodeRegTfbsClusteredV3 table ... OK
## Checking that required Columns are present ... 
## OK
## Prepare the 'metadata' data frame ... OK
## Make the AnnoDb object ...
```

```
          TXF_REINS_2020<-features(fdb)
          TXF_REINS_2020<-sort(TXF_REINS_2020)
          show(TXF_REINS_2020)
```

```
## GRanges object with 4638420 ranges and 5 metadata columns:
##                     seqnames      ranges strand |         bin       score
##                        <Rle>   <IRanges>  <Rle> | <character> <character>
##    ZBTB33               chr1 10074-10329      * |         585         354
##     CEBPB               chr1 10150-10413      * |         585         201
##      CTCF               chr1 16111-16390      * |         585         227
##      TAF1               chr1 29199-29688      * |         585         184
##     GABPA               chr1 29276-29591      * |         585         198
##       ...                ...         ...    ... .         ...         ...
##      CTCF chr22_kb663609_alt 54797-55092      * |         585         169
##       MAX chr22_kb663609_alt 54893-55064      * |         585         356
##   BHLHE40 chr22_kb663609_alt 56440-56735      * |         585         174
##       MAX chr22_kb663609_alt 56486-56665      * |         585         388
##      NFYB chr22_kb663609_alt 73496-73875      * |         585         383
##           sourceCount                   sourceIds                sourceScores
##           <character>                 <character>                 <character>
##    ZBTB33           2                     204,246                     354,138
##     CEBPB           1                         343                         201
##      CTCF           7 213,612,621,627,628,631,662 110,139,171,209,227,200,170
##      TAF1           1                         157                         184
##     GABPA           1                         180                         198
##       ...         ...                         ...                         ...
##      CTCF           6     603,630,665,667,673,688     169,161,147,140,159,142
##       MAX           1                         223                         356
##   BHLHE40           1                         423                         174
##       MAX           1                         223                         388
##      NFYB           2                     302,517                     369,383
##   -------
##   seqinfo: 235 sequences from hg19 genome; no seqlengths
```

Considering the amount of time used to download these genomic ranges it is highly recommended to save the data as a file in the working directory for future analyses.

```
 save(TXF_REINS_2020, file = "TXF_REINS_2020.Rdata")
```

### 3. **Input data**

Load of the data from the working directory that will be analyzed and some other data to make comparisons across diverse tissues or cell fates. The input data are character vectors with CpGs.

```
load("Inputdata_REINS_2020.Rdata", verbose = TRUE)
```

```
## Loading objects:
##   OB_array2_cpglist_hypo
##   OB_array2_cpglist_hyper
##   Adipose_tissue_hypocpgs
##   Adipose_tissue_hypercpgs
##   hPSC_tissue_hypocpgs
##   hPSC_tissue_hypercpgs
##   Thymus_tissue_hypocpgs
##   Thymus_tissue_hypercpgs
##   muscle_tissue_hypocpgs
##   muscle_tissue_hypercpgs
##   Pancreas_tissue_hypocpgs
##   Pancreas_tissue_hypercpgs
```

### 4. **txf4cpg function**

This is the main function of the script and has been optimized to be fast overlapping CpGs with TFs. Any modification in its code would involve a substantial increase in the calculation time.

```
  txf4cpg<-function (x){
          cov_matrix<-as.matrix( countOverlaps(TXF_REINS_2020,InfiniumMethylation[ x ]))
          split_cov_matrix<-split(cov_matrix, rownames(cov_matrix))
          return(as.matrix(lapply(split_cov_matrix,sum)))
          }
```

### 5. **Overlapping calculation**

- #### Tidy up the input data

  We put all the hypomethylated and hypermethylated sets of CpGs in different lists.

```
Hypo_Processes <- list(OB_array2_cpglist_hypo,Adipose_tissue_hypocpgs,hPSC_tissue_hypocpgs, Thymus_tissue_hypocpgs,      muscle_tissue_hypocpgs, Pancreas_tissue_hypocpgs)
Hyper_Processes <- list(OB_array2_cpglist_hyper,Adipose_tissue_hypercpgs,hPSC_tissue_hypercpgs, Thymus_tissue_hypercpgs, muscle_tissue_hypercpgs, Pancreas_tissue_hypercpgs)
```

- #### Calculation of the overlapping

  The results are storage and labeled in a matrix format for easy manipulation.

  First the overlapping calculation.

```
Binds_Ho <- matrix(unlist(list.cbind(lapply (Hypo_Processes,txf4cpg))), ncol = length(Hypo_Processes), byrow = FALSE)
Binds_Hr <- matrix(unlist(list.cbind(lapply (Hyper_Processes,txf4cpg))), ncol = length(Hyper_Processes), byrow = FALSE)
colnames(Binds_Ho)<-c("OB-Ho","Adip-Ho","PSC-Ho","Thym-Ho","Musc-Ho","Panc-Ho" )#short names to make better plots
colnames(Binds_Hr)<-c("OB-Hr","Adip-Hr","PSC-Hr","Thym-Hr","Musc-Hr","Panc-Hr")#short names to make better plots
row.names(Binds_Ho)<-row.names(list.cbind(lapply (Hypo_Processes,txf4cpg)))
row.names(Binds_Hr)<-row.names(list.cbind(lapply (Hyper_Processes,txf4cpg)))
head(Binds_Ho)
```

```
##        OB-Ho Adip-Ho PSC-Ho Thym-Ho Musc-Ho Panc-Ho
## ARID3A    81      19      7      83      31     184
## ATF1      45      13      1     104      25      28
## ATF2      81      37     50     563      41      52
## ATF3     141      23      7      70      52      69
## BACH1     27       5     16      21      12       9
## BATF      64      15      4     264      31      35
```

```
head(Binds_Hr)
```

```
##        OB-Hr Adip-Hr PSC-Hr Thym-Hr Musc-Hr Panc-Hr
## ARID3A     6       4     65      42       8      10
## ATF1       6       1     47       7       0       3
## ATF2      13      12     67      99      18      75
## ATF3       6       4     66      54       5      28
## BACH1      4       4     17      59      11      27
## BATF       7       0     37       8       1       7
```

Second the non overlapping calculation.

```
Not_Binds_Ho<-(sweep(Binds_Ho, 2, unlist(lapply (Hypo_Processes,length), use.names=FALSE), FUN ='-'))*(-1)
Not_Binds_Hr<-(sweep(Binds_Hr, 2, unlist(lapply (Hyper_Processes,length), use.names=FALSE), FUN ='-'))*(-1)
colnames(Not_Binds_Ho)<-colnames(Binds_Ho)
colnames(Not_Binds_Hr)<-colnames(Binds_Hr)
row.names(Not_Binds_Ho)<-row.names(Binds_Ho)
row.names(Not_Binds_Ho)<-row.names(Binds_Hr)
colnames(Not_Binds_Ho)<-gsub("-Ho", "-Ho_NB", colnames(Not_Binds_Ho))
colnames(Not_Binds_Hr)<-gsub("-Hr", "-Hr_NB", colnames(Not_Binds_Hr))
head(Not_Binds_Ho)
```

```
##        OB-Ho_NB Adip-Ho_NB PSC-Ho_NB Thym-Ho_NB Musc-Ho_NB Panc-Ho_NB
## ARID3A     1903        307       409       3343        751       1948
## ATF1       1939        313       415       3322        757       2104
## ATF2       1903        289       366       2863        741       2080
## ATF3       1843        303       409       3356        730       2063
## BACH1      1957        321       400       3405        770       2123
## BATF       1920        311       412       3162        751       2097
```

```
head(Not_Binds_Hr)
```

```
##        OB-Hr_NB Adip-Hr_NB PSC-Hr_NB Thym-Hr_NB Musc-Hr_NB Panc-Hr_NB
## ARID3A      472        151      1504       1026        613       1191
## ATF1        472        154      1522       1061        621       1198
## ATF2        465        143      1502        969        603       1126
## ATF3        472        151      1503       1014        616       1173
## BACH1       474        151      1552       1009        610       1174
## BATF        471        155      1532       1060        620       1194
```

We storage together all the overlapping and not overlapping results for all the hypo/hypermethylation paired datasets.

```
Ho_Hr_Binds<-cbind(Binds_Ho,Binds_Hr,Not_Binds_Ho,Not_Binds_Hr)
head(Ho_Hr_Binds)
```

```
##        OB-Ho Adip-Ho PSC-Ho Thym-Ho Musc-Ho Panc-Ho OB-Hr Adip-Hr PSC-Hr
## ARID3A    81      19      7      83      31     184     6       4     65
## ATF1      45      13      1     104      25      28     6       1     47
## ATF2      81      37     50     563      41      52    13      12     67
## ATF3     141      23      7      70      52      69     6       4     66
## BACH1     27       5     16      21      12       9     4       4     17
## BATF      64      15      4     264      31      35     7       0     37
##        Thym-Hr Musc-Hr Panc-Hr OB-Ho_NB Adip-Ho_NB PSC-Ho_NB Thym-Ho_NB
## ARID3A      42       8      10     1903        307       409       3343
## ATF1         7       0       3     1939        313       415       3322
## ATF2        99      18      75     1903        289       366       2863
## ATF3        54       5      28     1843        303       409       3356
## BACH1       59      11      27     1957        321       400       3405
## BATF         8       1       7     1920        311       412       3162
##        Musc-Ho_NB Panc-Ho_NB OB-Hr_NB Adip-Hr_NB PSC-Hr_NB Thym-Hr_NB
## ARID3A        751       1948      472        151      1504       1026
## ATF1          757       2104      472        154      1522       1061
## ATF2          741       2080      465        143      1502        969
## ATF3          730       2063      472        151      1503       1014
## BACH1         770       2123      474        151      1552       1009
## BATF          751       2097      471        155      1532       1060
##        Musc-Hr_NB Panc-Hr_NB
## ARID3A        613       1191
## ATF1          621       1198
## ATF2          603       1126
## ATF3          616       1173
## BACH1         610       1174
## BATF          620       1194
```

### 6. **Data processing**

Calculation of the TFR (transcription factor relevance) metric. This metric is the percentage of CpGs overlapping each TXF for each list of CpGs associated to a given process.

```
TFR_Ho<-(sweep(Binds_Ho, 2, unlist(lapply (Hypo_Processes,length), use.names=FALSE), FUN ='/'))*100
TFR_Hr<-(sweep(Binds_Hr, 2, unlist(lapply (Hyper_Processes,length), use.names=FALSE), FUN ='/'))*100
colnames(TFR_Ho)<-colnames(Binds_Ho)
colnames(TFR_Hr)<-colnames(Binds_Hr)
row.names(TFR_Ho)<-row.names(Binds_Ho)
row.names(TFR_Hr)<-row.names(Binds_Hr)
head(TFR_Ho)
```

```
##           OB-Ho   Adip-Ho     PSC-Ho    Thym-Ho  Musc-Ho   Panc-Ho
## ARID3A 4.082661  5.828221  1.6826923  2.4226503 3.964194 8.6303940
## ATF1   2.268145  3.987730  0.2403846  3.0356100 3.196931 1.3133208
## ATF2   4.082661 11.349693 12.0192308 16.4331582 5.242967 2.4390244
## ATF3   7.106855  7.055215  1.6826923  2.0431991 6.649616 3.2363977
## BACH1  1.360887  1.533742  3.8461538  0.6129597 1.534527 0.4221388
## BATF   3.225806  4.601227  0.9615385  7.7057793 3.964194 1.6416510
```

```
head(TFR_Hr)
```

```
##            OB-Hr   Adip-Hr   PSC-Hr   Thym-Hr   Musc-Hr   Panc-Hr
## ARID3A 1.2552301 2.5806452 4.142766 3.9325843 1.2882448 0.8326395
## ATF1   1.2552301 0.6451613 2.995539 0.6554307 0.0000000 0.2497918
## ATF2   2.7196653 7.7419355 4.270236 9.2696629 2.8985507 6.2447960
## ATF3   1.2552301 2.5806452 4.206501 5.0561798 0.8051530 2.3313905
## BACH1  0.8368201 2.5806452 1.083493 5.5243446 1.7713366 2.2481266
## BATF   1.4644351 0.0000000 2.358190 0.7490637 0.1610306 0.5828476
```

We storage together all the TFRs for the hypomethylated and hypermethylated datasets.

```
HoHr_TFR<-cbind(TFR_Ho,TFR_Hr)
head(HoHr_TFR)
```

```
##           OB-Ho   Adip-Ho     PSC-Ho    Thym-Ho  Musc-Ho   Panc-Ho     OB-Hr
## ARID3A 4.082661  5.828221  1.6826923  2.4226503 3.964194 8.6303940 1.2552301
## ATF1   2.268145  3.987730  0.2403846  3.0356100 3.196931 1.3133208 1.2552301
## ATF2   4.082661 11.349693 12.0192308 16.4331582 5.242967 2.4390244 2.7196653
## ATF3   7.106855  7.055215  1.6826923  2.0431991 6.649616 3.2363977 1.2552301
## BACH1  1.360887  1.533742  3.8461538  0.6129597 1.534527 0.4221388 0.8368201
## BATF   3.225806  4.601227  0.9615385  7.7057793 3.964194 1.6416510 1.4644351
##          Adip-Hr   PSC-Hr   Thym-Hr   Musc-Hr   Panc-Hr
## ARID3A 2.5806452 4.142766 3.9325843 1.2882448 0.8326395
## ATF1   0.6451613 2.995539 0.6554307 0.0000000 0.2497918
## ATF2   7.7419355 4.270236 9.2696629 2.8985507 6.2447960
## ATF3   2.5806452 4.206501 5.0561798 0.8051530 2.3313905
## BACH1  2.5806452 1.083493 5.5243446 1.7713366 2.2481266
## BATF   0.0000000 2.358190 0.7490637 0.1610306 0.5828476
```

With this metric it is possible to make comparisons across different TFs and CpG datasets. To visualize these comparisons we create different heatmaps clustering the results.

```
heatmap(TFR_Ho,margins = c(5,3) ,cexRow = 0.38,cexCol = 0.8) #plot for the hypomethylated CpGs
```

```
heatmap(TFR_Hr,margins = c(5,3) ,cexRow = 0.38,cexCol = 0.8) #plot for the hypermethylated CpGs
```

```
heatmap(HoHr_TFR,margins = c(5,3) ,cexRow = 0.38,cexCol = 0.8)#plot for the hypo- and hypermethylated CpGs
```

```
heatmap(t(TFR_Ho),margins= c(5,3) ,cexRow = 0.38,cexCol = 0.8)
```

```
heatmap(t(TFR_Hr),margins= c(5,3) ,cexRow = 0.38,cexCol = 0.8)
```

```
heatmap(t(HoHr_TFR),margins= c(5,3) ,cexRow = 0.38,cexCol = 0.8)
```

To visualize the different TFRs across diverse datasets for a given TF we create a barplot.

```
TXF2plot<-"ZEB1"# Input here the search term
numberofTXF<-match(TXF2plot,row.names(HoHr_TFR))
barplot( HoHr_TFR[  numberofTXF, order(colnames(HoHr_TFR))],
              col=rep(c("white","black"),times= length(colnames(HoHr_TFR))/2), 
                main=TXF2plot, ylab="Percentage %",las=2 )
```

In order to explore the relationship between the hipomethylated TFR and the hypermethylated profile for each TF we calculate the RRT metric. This metric further helps to compare across TFs and CpG datasets.

```
RRT<-TFR_Ho/TFR_Hr
colnames(RRT)<-gsub("-Ho", "", colnames(RRT))
RRT[is.na(RRT)] <- 0
RRT[is.infinite(RRT)] <- 0
head(RRT)
```

```
##              OB      Adip        PSC       Thym       Musc       Panc
## ARID3A 3.252520 2.2584356 0.40617604  0.6160454  3.0772059 10.3651032
## ATF1   1.806956 6.1809816 0.08024755  4.6314736  0.0000000  5.2576610
## ATF2   1.501163 1.4660020 2.81465270  1.7727892  1.8088235  0.3905691
## ATF3   5.661794 2.7338957 0.40002185  0.4040994  8.2588235  1.3881835
## BACH1  1.626260 0.5943252 3.54977376  0.1109561  0.8663102  0.1877736
## BATF   2.202765 0.0000000 0.40774428 10.2872154 24.6176471  2.8166041
```

WARNING!! in order to plot correctly NA, -Inf, and Inf were substituted by 0. This means the loss of certain data. As we did for the percentages we create heatmap to compare the different datasets.

```
heatmap(RRT,margins = c(5,3) ,cexRow = 0.38,cexCol = 0.8)
```

```
heatmap(t(RRT),margins= c(5,3) ,cexRow = 0.38,cexCol = 0.8)
```

To visualize the different RRTs across diverse datasets for a given TF we create a barplot.

```
TXF2plot<-"ZEB1"# Input the search term
numberofTXF<-match(TXF2plot,row.names(RRT))
barplot( RRT[numberofTXF, ],
        col=rep(c("red"),times= length(colnames(RRT))), 
        main=TXF2plot, ylab="RRT",las=2 )
```

To compare TFs RRTs in a given dataset we also create an ordered barplot.

```
# all TXFs Ordered according to the RRT values
Fateplot<-"OB"# Input the search term
fate4TXF<-match(Fateplot,colnames(RRT))
barplot( RRT[order(RRT[,fate4TXF]),fate4TXF],
col=rep(c("red"),times= length(colnames(RRT))), 
main=Fateplot, ylab="RRT",las=2, cex.names = 0.5 )
```

To achieve a better visualization of the results we make a logarithmic transformation of the data.

```
log_RRT<-log2(RRT)
head(log_RRT)
```

```
##               OB       Adip       PSC       Thym       Musc       Panc
## ARID3A 1.7015580  1.1753238 -1.299823 -0.6988915  1.6216210  3.3736626
## ATF1   0.8535611  2.6278360 -3.639399  2.2114713       -Inf  2.3944211
## ATF2   0.5860808  0.5518871  1.492957  0.8260210  0.8550517 -1.3563503
## ATF3   2.5012593  1.4509582 -1.321849 -1.3072180  3.0459363  0.4731982
## BACH1  0.7015580 -0.7506757  1.827727 -3.1719391 -0.2070445 -2.4129338
## BATF   1.1393156       -Inf -1.294263  3.3627806  4.6216210  1.4939568
```

```
log_RRT[is.infinite(log_RRT)] <- 0
```

WARNING!! in order to plot correctly NA, -Inf, and Inf were substituted by 0. We create heatmap to compare the different datasets.

```
heatmap(log_RRT,margins = c(5,3) ,cexRow = 0.38,cexCol = 0.8)
```

```
heatmap(t(log_RRT),margins= c(5,3) ,cexRow = 0.38,cexCol = 0.8)
```

To visualize the different log-RRTs across diverse datasets for a given TF we create a barplot.

```
TXF2plot<-"ZEB1"# Input the search term
numberofTXF<-match(TXF2plot,row.names(log_RRT))
barplot( log_RRT[numberofTXF, ],
        col=rep(c("red"),times= length(colnames(log_RRT))), 
        main=TXF2plot, ylab="log_RRT",las=2 )
```

To compare TFs log-RRTs in a given dataset we also create an ordered barplot.

```
Fateplot<-"OB"# Input the search term
fate4TXF<-match(Fateplot,colnames(log_RRT))
barplot( log_RRT[order(log_RRT[,fate4TXF]),fate4TXF],
        col=rep(c("red"),times= length(colnames(log_RRT))), 
         main=Fateplot, ylab="log_RRT",las=2, cex.names = 0.5)
```

### 7. **Statistics**

In order to determine whether the observed enrichment of hypermethylated or hipomethylated CpGs for each TF is statistically different from the expected enrichment, we calculate the latter one determining the TF overlapping with all the CpGs studied in the array.

```
cov<-(countOverlaps(TXF_REINS_2020,InfiniumMethylation))
cov_matrix<-as.matrix(cov)
split_cov_matrix<-split(cov_matrix, rownames(cov_matrix))
Number_binding_TXF<-as.matrix(lapply(split_cov_matrix,sum))
Binds_Array<-as.matrix(unlist(Number_binding_TXF[,1]))
Not_Binds_Array<-(Binds_Array-length(InfiniumMethylation))*-1
Array_profile<-cbind(Binds_Array,Not_Binds_Array)
colnames(Array_profile)<-c("Array-Bind", "Array-NB")
head(Array_profile)
```

```
##        Array-Bind Array-NB
## ARID3A      14428   472745
## ATF1        14737   472436
## ATF2        30310   456863
## ATF3        25934   461239
## BACH1       16321   470852
## BATF         5317   481856
```

To calculate the statistical significance of the differences observed between the observed enrichment and the expected enrichment we create the function fisher.txf.

```
   fisher.txf<-function(x,y){
          numberofTXF<-match(x,row.names(Ho_Hr_Binds))
          Poch<-Ho_Hr_Binds[numberofTXF,grep(y, colnames(Ho_Hr_Binds))]
          Convictions <-matrix(c(   Poch[1],
                                    Poch[2], 
                                    Array_profile[numberofTXF,1], 
                                    Array_profile[numberofTXF,2]),                       
                                    nrow = 2,
                                    dimnames =
                                    list(c("TXF-Bind", "TXF-Notbind"),
                                      c("Observed", "array")))
          Convictions_stats<-fisher.test(Convictions)
          
          return( Convictions_stats$p.value)
        }
```

Example: this function needs two arguments: the Tf as character and the name of the CpG list as character.

```
fisher.txf("ZEB1","OB-Ho")
```

```
## [1] 6.306998e-13
```

This function determines the p-values (fisher text) for each TF in a given list of CPGs.

```
 fisher.all<-function(Loo){lapply(row.names(Ho_Hr_Binds), fisher.txf,y=Loo)}
```

Example for all the TFs in a given list.

```
fisher_dataset<-as.matrix(fisher.all("OB-Ho"))
rownames(fisher_dataset)<-rownames(Ho_Hr_Binds)
head(fisher_dataset)
```

```
##        [,1]        
## ARID3A 0.005211407 
## ATF1   0.04852908  
## ATF2   3.933882e-05
## ATF3   0.0007716551
## BACH1  4.223713e-08
## BATF   1.045641e-13
```

For all the TFs and for all the list of Cpgs at the same time.

```
Fisher_Binds<-sapply(colnames(Ho_Hr_Binds[,1:(length(colnames(Ho_Hr_Binds))/2)]),fisher.all)
rownames(Fisher_Binds)<-rownames(Ho_Hr_Binds)
head(Fisher_Binds)
```

```
##        OB-Ho        Adip-Ho      PSC-Ho       Thym-Ho       Musc-Ho     
## ARID3A 0.005211407  0.00503381   0.1462276    0.0682105     0.1117575   
## ATF1   0.04852908   0.327549     7.585492e-05 0.9600616     0.7534256   
## ATF2   3.933882e-05 0.0004906363 9.795843e-06 1.726396e-95  0.2989139   
## ATF3   0.0007716551 0.1727661    0.0002658176 5.081338e-22  0.1100749   
## BACH1  4.223713e-08 0.08691941   0.5835863    3.774738e-27  0.00269479  
## BATF   1.045641e-13 4.327919e-06 1            3.252418e-129 1.826287e-09
##        Panc-Ho      OB-Hr        Adip-Hr    PSC-Hr       Thym-Hr     
## ARID3A 2.18026e-36  0.02141075   1          0.008836202  0.06993362  
## ATF1   4.450813e-07 0.02180032   0.09682157 1            1.342987e-07
## ATF2   4.706179e-16 0.0006104852 0.4044709  0.0009480496 0.0001035415
## ATF3   6.33059e-06  5.37157e-06  0.1514283  0.04852274   0.7846881   
## BACH1  1.144394e-20 0.0007823251 0.8222725  1.341745e-08 0.000238119 
## BATF   0.02092912   0.3757102    0.4225297  2.440725e-05 0.3734974   
##        Musc-Hr      Panc-Hr     
## ARID3A 0.01203163   5.837594e-07
## ATF1   1.10147e-08  2.196955e-12
## ATF2   0.0002325815 0.9523001   
## ATF3   1.682078e-09 4.223104e-07
## BACH1  0.02510231   0.03615149  
## BATF   0.01806894   0.09450261
```

### 7. **REINS output**

To storage the output of REINS script in a exportable format we create a .txt file that will we located at the work space.

```
write.table(cbind(Array_profile,Ho_Hr_Binds,Fisher_Binds, HoHr_TFR, RRT, log_RRT), file = "REINS_output.txt", append = FALSE, quote = TRUE, sep = " ",
                    eol = "\n", na = "NA", dec = ".", row.names = TRUE,
                    col.names = NA, qmethod = c("escape", "double"))
```
