# Supplementary material for: DNA methylation analysis identifies key transcription factors involved in mesenchymal stem cell osteogenic differentiation
Source: Biol Res. 2023 Mar 8;56:9. doi: 10.1186/s40659-023-00417-6 (PMC9996951; doi:10.1186/s40659-023-00417-6)
Supplement: Supplementary file 2 — Additional file 2: Model design. A computational model of Bmp2 signalling was constructed to evaluate ALP induction in the context of ZEB TFs activity. [file 40659_2023_417_MOESM2_ESM.docx]

**Computational modelling**

A computational model of Bmp2 signalling was constructed to evaluate ALP induction in the context of ZEB TFs activity. A simplified diagram shows the main features of the model including the role of Zeb1 and Zeb2 in the Bmp2 pathway (figure SD-1). Full details of the model are given in the Model Assumptions section. We ran the model using stochastic simulation as this allows for random fluctuations in protein levels and in interactions between proteins. Each simulation gives a different trajectory and so can account for intra-cellular variability. Therefore we ran the model 100 times over a time period of 21 days (virtual time).

The model was constructed in the Systems Biology Markup Language (SBML)[1] using the Python tool SBML shorthand [2]. SBML is a modelling standard, which allows models to be readily modified and extended as required. The model has been deposited in the public database Biomodels [3] and assigned the identifier MODEL1702220000. Stochastic simulations were carried out in COPASI [4] during the model development stage and then multiple simulations were carried out on a computer cluster using code developed by Newcastle University [5]. All simulations were carried out using the Gillespie direct algorithm [6]. Simulation results were analysed in the R statistical programming language and graphs were constructed with ggplot2 package [7]. Network figures were constructed in CellDesigner [8] using the standard Systems Biology Graphical Notation (SBGN) [9].

The model output shows that in all simulations both Zeb1 and Zeb2 increase rapidly up to day 3 and then levels remain fairly constant but with random fluctuations especially in levels of Zeb2 (Fig SD-2 A). The Zeb1/Zeb2 ratio also increases up to day 3 in all simulations and then fluctuates but with no further upward trend (Fig SD-2 B). The initial increase in the Zeb1/Zeb2 ratio indicates that Zeb1 increases far more than Zeb2 in the first three days. ALP mRNA levels also increase over the first three days, following a similar pattern to Zeb1/2 (Fig SD-2 A). The mean and standard deviation of 100 runs is shown in (Fig SD-2 A), which shows that there are no significant changes in Zeb protein or ALP mRNA after day 3.


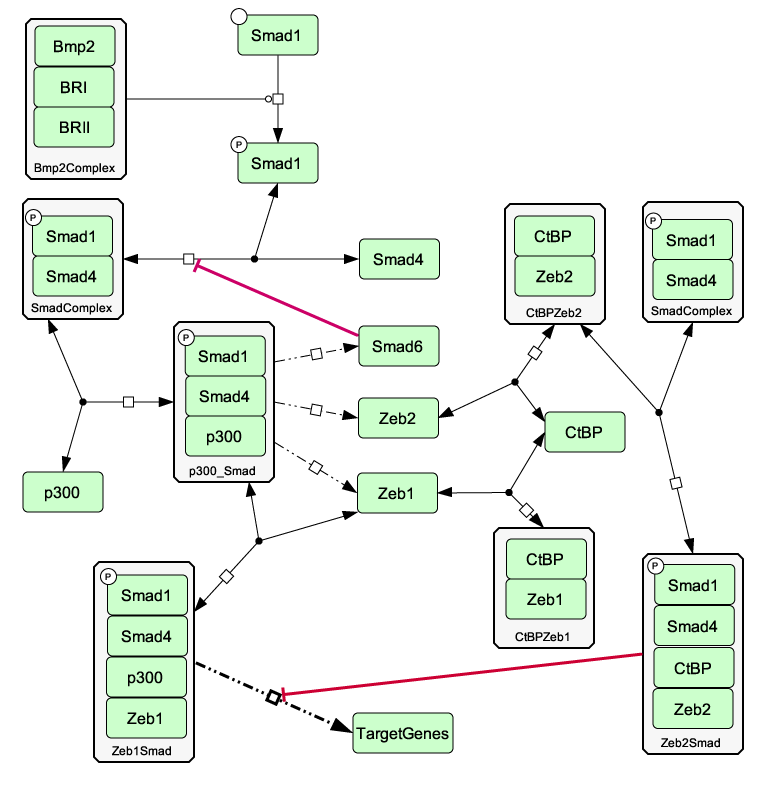


Reaction

Transcription

Activation

Inhibition

Phosphorylation

**P**

**Figure SD-1. Diagram showing key components of the model:** Bmp2 signalling leads to phosphorylation of Smad1 which forms complex with Smad4 and p300 to upregulate Zeb1, Zeb2 and Smad6. The Smad/p300 complex also binds to Zeb1 leading to increased transcription of target genes. CtBP binds to Zeb2 which then forms a complex with Smad1/4 to inhibit transcription of Zeb1 target genes. CtBP also binds to Zeb1 to inhibit its activity. The network was created in CellDesigner [[1](#_ENREF_1)].


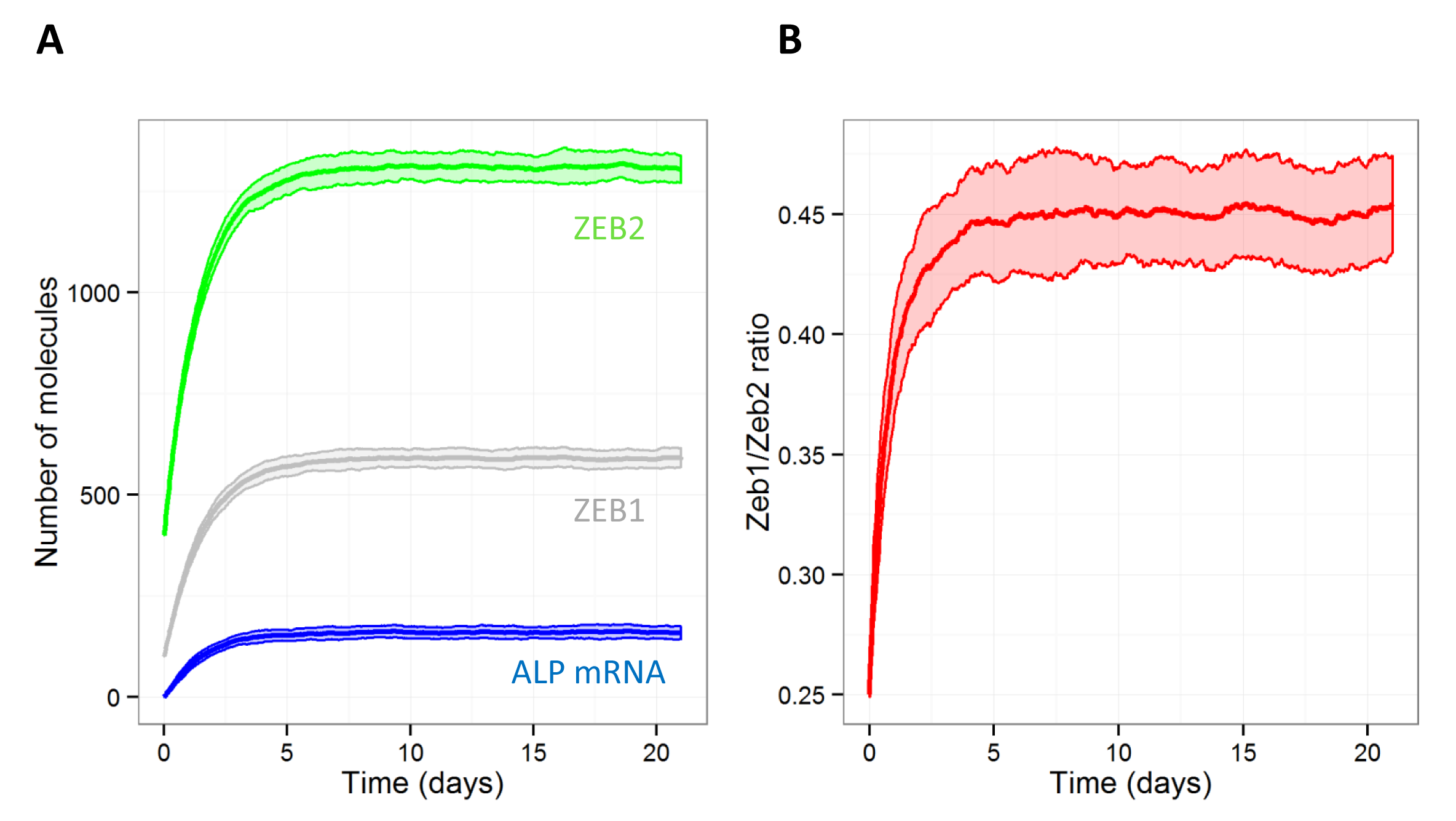


**Figure SD-2. Output from 100 simulations:** The mean (solid line) and standard deviation (shaded area) of 100 stochastic simulations. (A) Zeb1, Zeb2 protein, and ALP mRNA; (B) Zeb1/Zeb2 ratio.

**Additional file Information**

**Model Assumptions**

**BMP2 signalling**

A simple model of the pathway, which can be extended, if required, was constructed. The model included the following steps.

1. Bmp2 is activated and binds (reversibly) to BRI/BRII receptor complex.
2. This complex phosphorylates Smad1.
3. Phosphorylated Sma13 binds (reversibly) to Smad 4.
4. Smad1/Smad4 complex binds to p300 to activate transcription of genes (ALP, cJun, Smad6, Zeb1).
5. Smad1/Smad4 complex also binds to p300/Zeb1 complex which results in enhanced transcription of the above genes and also transcription of Zeb2.
6. Smad6 binds to Smad1_P, which prevents Smad4 binding. Therefore Smad6 provides a negative feedback loop to switch off Bmp2 signalling.

**Role of Zeb1 in Bmp2 signalling**

The model included the following assumptions regarding the role of Zeb1 in Bmp2 signalling:

1. Zeb1 binds to CtBP to form a complex. This complex interacts with Smad1/4 and binds to gene promoters to inhibit transcription. Inhibited targets are ALP, cJun and Zeb1.
2. P300 binds to Zeb1 and so competes with CtBP for binding. We assume that this binding rate is 10x stronger than the CtBP/Zeb1 binding rate.
3. p300/Zeb1 complex binds to Smad1/4 and then binds to promoters to enhance transcription. Target genes included are ALP, Smad6, cJun, Zeb1 and Zeb2.
4. Zeb1 is degraded with a half-life of 24 h.

**Role of Zeb2 in Bmp2 signalling**

The model included the following assumptions regarding the role of Zeb2 in Bmp2 signalling:

1. Zeb2 binds to CtBP to form a complex. This complex binds to Smad1/4 and then binds to promoters to inhibit transcription. Inhibited targets are ALP, cJun and Zeb1.
2. Zeb2/CtBP/Smad1/4 has stronger affinity for DNA than Zeb1/p300/Smad1/4.
3. Transcription of Zeb2 is either via cJun or p300/Zeb1/Smad1/4.
4. Zeb2 is degraded with a half-life of 24 h.

Full details of model species are given Table A. Details of reactions and parameter values are contained in the SBML file which has been deposited in BioModels [[2](#_ENREF_2)] and assigned the identifier: MODEL1702220000.

**Table A List of model species and their HUGO names**

| **Species id** | **Description** | **Database term** | **Initial amount** |
| --- | --- | --- | --- |
| ALP_gene | Promoter region of ALP gene | [HGNC:438](http://www.genenames.org/cgi-bin/gene_symbol_report?hgnc_id=438) | 2 |
| ALP_­mRNA | mRNA of ALP | [HGNC:438](http://www.genenames.org/cgi-bin/gene_symbol_report?hgnc_id=438) | 0 |
| Bmp2_A | Activated Bmp2 | [HGNC:1069](http://www.genenames.org/cgi-bin/gene_symbol_report?hgnc_id=HGNC:1069) | 190 |
| Bmp2_BRI_BRII | Bmp2 bound to receptor complex |  | 0 |
| Bmp2_I | Inactive Bmp2 |  | 10 |
| BRI | Type 1 Bmp2 receptor (BMPR1A) | [HGNC:1076](http://www.genenames.org/cgi-bin/gene_symbol_report?hgnc_id=HGNC:1076) | 500 |
| BRII | Type 2 Bmp2 receptor (BMPR2) | [HGNC:1078](http://www.genenames.org/cgi-bin/gene_symbol_report?hgnc_id=HGNC:1078) | 500 |
| BRI_BRII | Bmp2 receptor complex |  | 0 |
| cJun | AP1 transcription factor (JUN) | [HGNC:6204](http://www.genenames.org/cgi-bin/gene_symbol_report?hgnc_id=HGNC:6204) | 0 |
| cJun_dimer | cJun dimer |  | 0 |
| cJun_dimer_cJungene | cJun_dimer bound to cJun promoter |  | 0 |
| cJun_dimer_Zeb2_gene | cJun_dimer bound to Zeb2 promoter |  | 0 |
| cJun_gene | Promoter region of cJun |  | 2 |
| cJun_P | Phosphorylated cJun |  | 0 |
| CtBP | C-terminal binding protein 1 (CTBP1) | [HGNC:2494](http://www.genenames.org/cgi-bin/gene_symbol_report?hgnc_id=HGNC:2494) | 100 |
| CtBP_Zeb1 | CtBP bound to Zeb1 |  | 0 |
| CtBP_Zeb1_Smad1_P_Smad4 | CtBP_Zeb1 bound to Smad1/4 complex |  | 0 |
| CtBP_Zeb1_Smad1_P_Smad4_ALP_gene | CtBP_Zeb1_Smad1_P_Smad4 bound to ALP promoter |  | 0 |
| CtBP_Zeb1_Smad1_P_Smad4_  cJun_gene | CtBP_Zeb1_Smad1_P_Smad4 bound to cJun promoter |  | 0 |
| CtBP_Zeb1_Smad1_P_Smad4_  Zeb1_gene | CtBP_Zeb1_Smad1_P_Smad4 bound to Zeb1 promoter |  | 0 |
| CtBP_Zeb2 | CtBP bound to Zeb2 |  | 0 |
| CtBP_Zeb2_Smad1_P_Smad4 | CtBP_Zeb2 bound to Smad complex |  | 0 |
| CtBP_Zeb2_Smad1_P_Smad4_ALP_gene | CtBP_Zeb2_Smad1_P_Smad4 bound to ALP promoter |  | 0 |
| CtBP_Zeb2_Smad1_P_Smad4_cJun_gene | CtBP_Zeb2_Smad1_P_Smad4 bound to cJun promoter |  | 0 |
| CtBP_Zeb2_Smad1_P_Smad4_  Zeb1_gene | CtBP_Zeb2_Smad1_P_Smad4 bound to Zeb1 promoter |  | 0 |
| p300 | Histone acetyltransferase p300 | [HGNC:3373](http://www.genenames.org/cgi-bin/gene_symbol_report?hgnc_id=HGNC:3373) | 100 |
| p300_Smad1_P_Smad4 | p300 bound to Smad complex |  | 0 |
| p300_Smad1_P_Smad4_  ALP_gene | p300_Smad1_P_Smad4  bound to ALP promoter |  | 0 |
| p300_Smad1_P_Smad4_  cJun_gene | p300_Smad1_P_Smad4  bound to cJun promoter |  | 0 |
| p300_Smad1_P_Smad4_  Zeb1_gene | p300_Smad1_P_Smad4  bound to Zeb1 promoter |  | 0 |
| p300_Zeb1 | p300 bound to Zeb1 protein |  | 0 |
| p300_Zeb1_Smad1_P_Smad4 | p300_Zeb1 bound to Smad complex |  | 0 |
| p300_Zeb1_Smad1_P_Smad4_ALP_gene | p300_Zeb1_Smad1_P_Smad4 bound to ALP promoter |  | 0 |
| p300_Zeb1_Smad1_P_Smad4_  cJun_gene | p300_Zeb1_Smad1_P_Smad4 bound to cJun promoter |  | 0 |
| p300_Zeb1_Smad1_P_Smad4_Zeb1_gene | p300_Zeb1_Smad1_P_Smad4 bound to Zeb1 promoter |  | 0 |
| p300_Zeb1_Smad1_P_Smad4_Zeb2_gene | p300_Zeb1_Smad1_P_Smad4 bound to Zeb2 promoter |  | 0 |
| Smad1 | Smad1 protein | [HGNC:6767](http://www.genenames.org/cgi-bin/gene_symbol_report?hgnc_id=HGNC:6767) | 600 |
| Smad1_P | Phosphorylated Smad1 |  | 0 |
| Smad4 | Smad4 protein | [HGNC:6770](http://www.genenames.org/cgi-bin/gene_symbol_report?hgnc_id=HGNC:6770) | 600 |
| Smad1_P_Smad4 | Smad1_P bound to Smad4 |  | 0 |
| Smad1_P_Smad6 | Smad1_P bound to Smad6 |  | 0 |
| Smad6 | Smad6 protein | [HGNC:6772](http://www.genenames.org/cgi-bin/gene_symbol_report?hgnc_id=HGNC:6772) | 0 |
| Zeb1 | Zeb1 protein | [HGNC:11642](http://www.genenames.org/cgi-bin/gene_symbol_report?hgnc_id=HGNC:11642) | 100 |
| Zeb1_gene | Promoter of Zeb1 |  | 2 |
| Zeb2 | Zeb2 protein | [HGNC:14881](http://www.genenames.org/cgi-bin/gene_symbol_report?hgnc_id=HGNC:14881) | 400 |
| Zeb2_gene | Promoter of Zeb2 |  | 2.0 |

**References**

1. Funahashi, A., et al., *CellDesigner 3.5: A Versatile Modeling Tool for Biochemical Networks.* 1. Hucka, M., et al., *The systems biology markup language (SBML): a medium for representation and exchange of biochemical network models.* Bioinformatics, 2003. **19**(4): p. 524-531.

2. Wilkinson, D.J., *Stochastic Modelling for Systems Biology*. 2006: Chapman & Hall/CRC Press.

3. Li, C., et al., *BioModels Database: An enhanced, curated and annotated resource for published quantitative kinetic models.* BMC Syst Biol, 2010. **4**: p. 92.

4. Hoops, S., et al., *COPASI--a COmplex PAthway SImulator.* Bioinformatics, 2006. **22**(24): p. 3067-74.

5. Gillespie, C.S., et al., *Tools for the SBML community.* Bioinformatics, 2006. **22**(5): p. 628-629.

6. Gillespie, D.T., *Exact stochastic simulation of coupled chemical reactions.* The Journal of Physical Chemistry, 1977. **31**: p. 2340-2361.

7. Wickham, H., *ggplot2: elegant graphics for data analysis*. 2009: Springer.

8. Funahashi, A., et al., *CellDesigner 3.5: A versatile modeling tool for biochemical networks.* Proceedings of the IEEE, 2008. **96**(8): p. 1254-1265.

9. Le Novere, N., et al., *The Systems Biology Graphical Notation.* Nat Biotechnol, 2009. **27**(8): p. 735-41.
