# Supplementary figures and images for: DNA methylation analysis identifies key transcription factors involved in mesenchymal stem cell osteogenic differentiation
Source: Biol Res. 2023 Mar 8;56:9. doi: 10.1186/s40659-023-00417-6 (PMC9996951; doi:10.1186/s40659-023-00417-6)

A

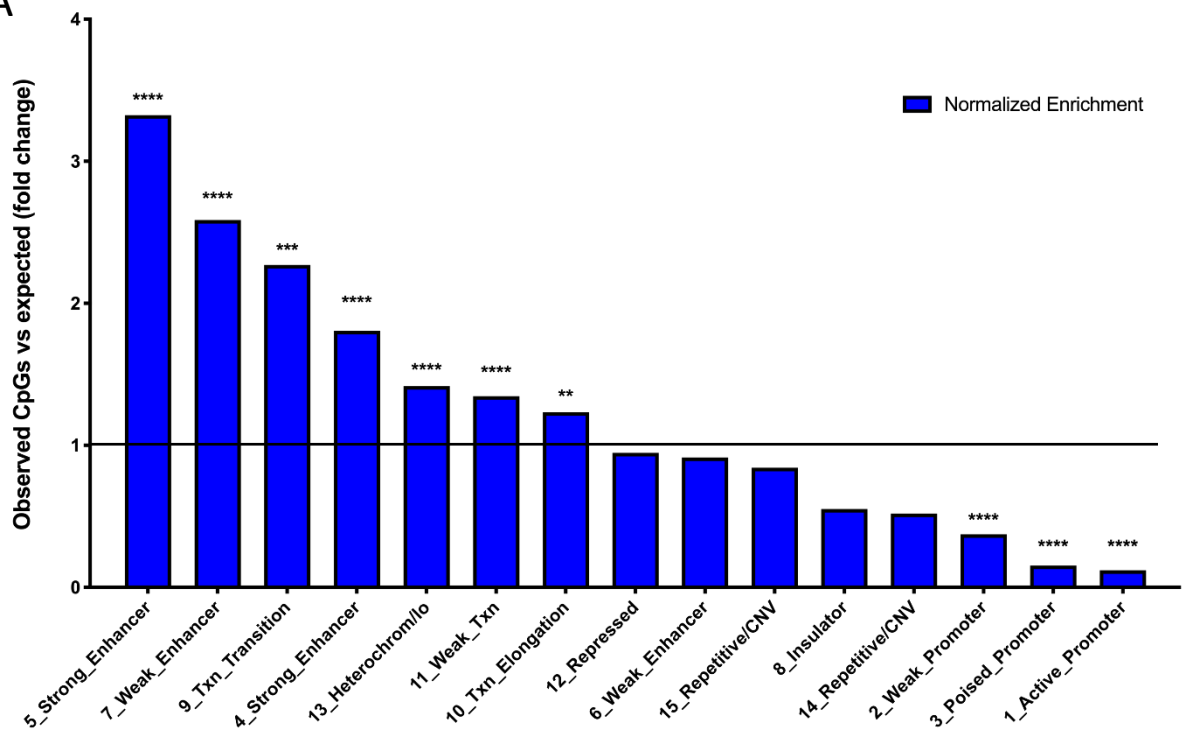

B

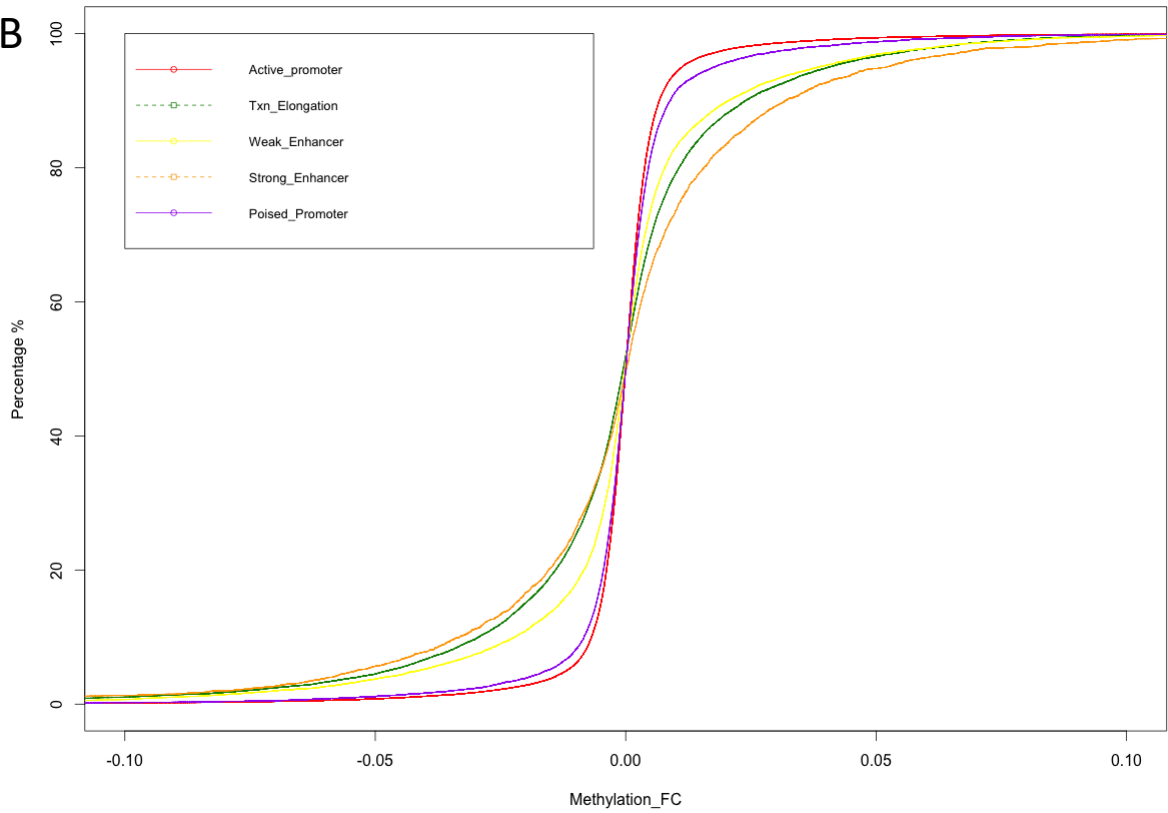

Supplement: Supplementary file 6 — Additional file 6: Figure S1. Observed vs expected ratio of CpGs enrichment among the different chromatin states and their cumulative methylation profile in MSCs. A) The bars represent the ratio of the percentage of differently methylated CpGs vs the percentage of the total CpGs studied for each chromatin state. B) Cumulative plot of the methylation of the CpGs overlapping top enriched (Strong_ Enhancer, Weak_ Enhancer, and Txn_Transition) and depleted chromatin states (Active_promoter and Poised_ Promoter). Y axis: cumulative percentage of CpGs overlapping to each chromatin state. X axis: fold change methylation MSCs vs Osteoblasts. [file 40659_2023_417_MOESM6_ESM.pdf]

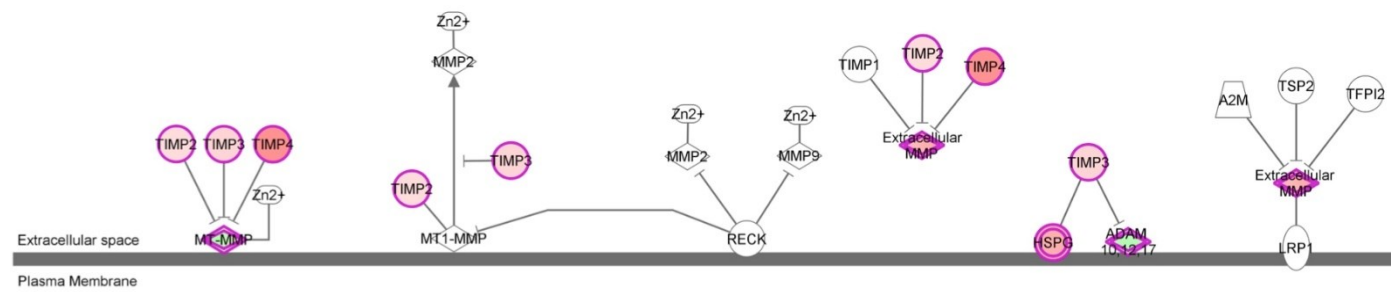

**Inhibition of MMPs**  
 $p=0.00142538$

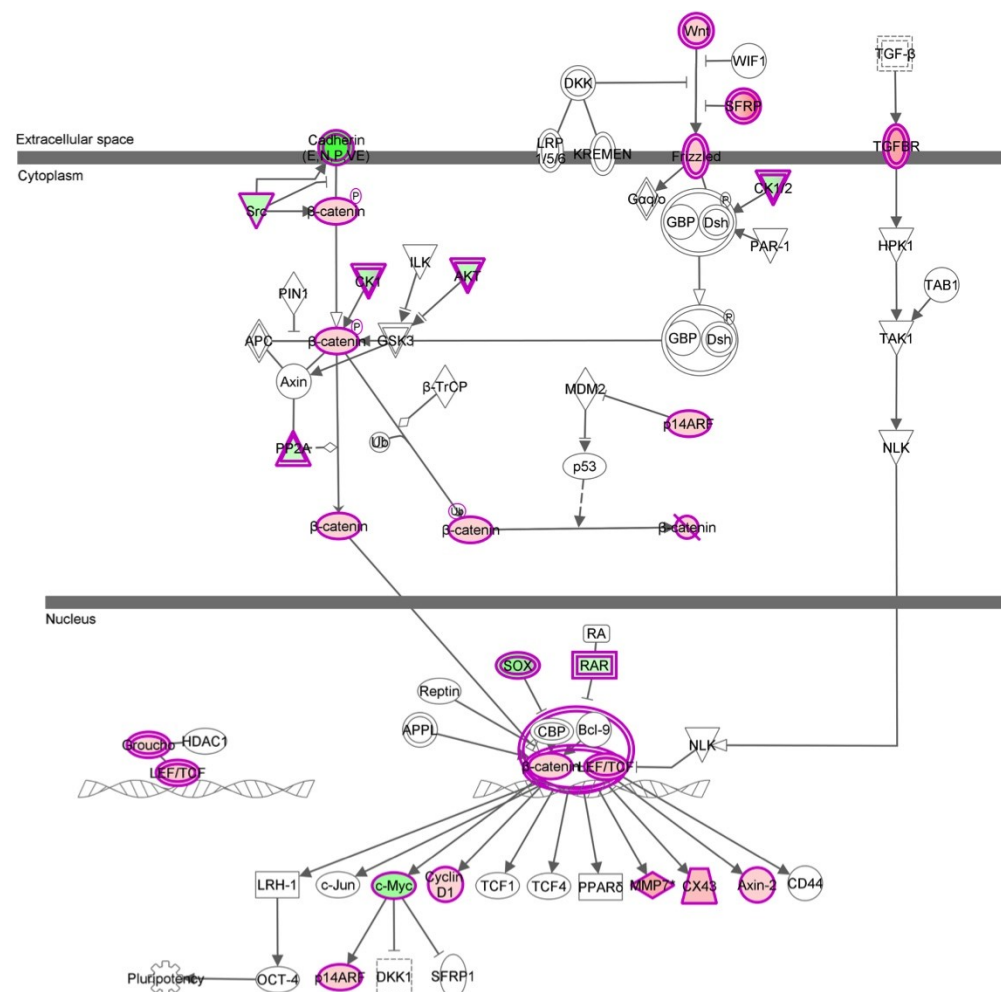

**WNT pathway activation**  
 $p=0.000645654$

Supplement: Supplementary file 7 — Additional file 7: Figure S2. Output of ingenuity pathway analysis. Representation of the significant enrichment on ‘inhibition of MMPs’ as well as ‘WNT pathway activation’. [file 40659_2023_417_MOESM7_ESM.pdf]

A

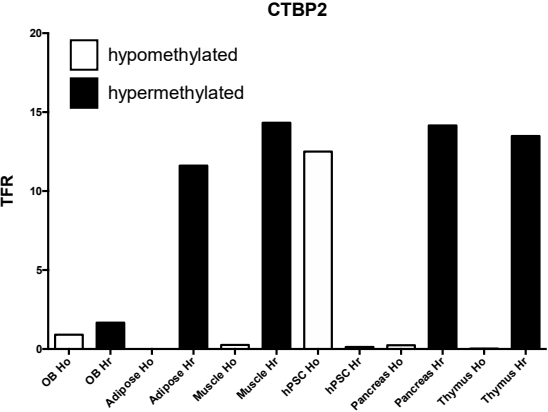

B

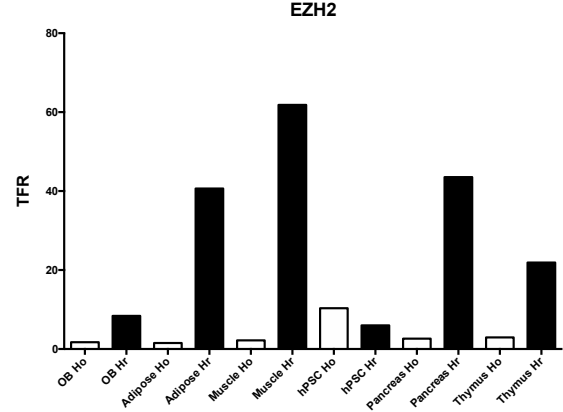

C

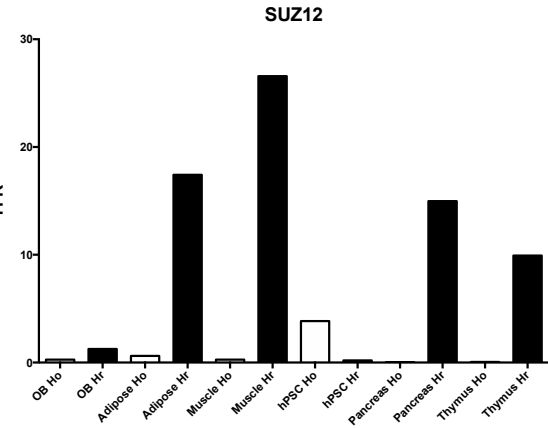

D

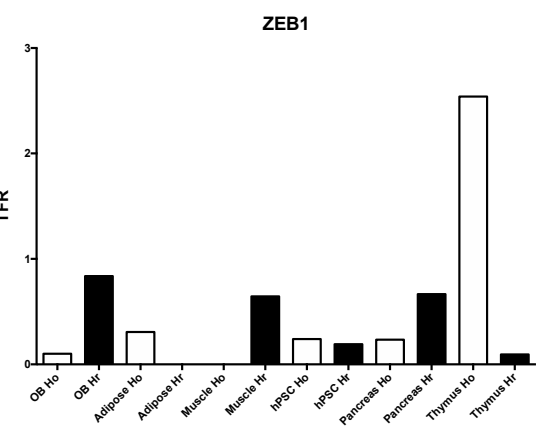

Supplement: Supplementary file 8 — Additional file 8: Figure S3. Validation of REINS algorithm. Transcription factor relevance (TFR) metric for hypo (Ho)- and hypermethylated (Hr) CpGs associated to MSCs differentiation to osteoblasts (OB), to different tissues (muscle, adipose tissue, pancreas, thymus) and to human pluripotent stem cells (hPSC). A) TFR for the repressor TF CTBP2. B) TFR for the TF EZH2 a member of the polycomb repressive complex 2 (PRC2). C) TFR for the TF SUZ12 a member of the polycomb repressive complex 2 (PRC2). D) TFR for the TF ZEB1. [file 40659_2023_417_MOESM8_ESM.pdf]

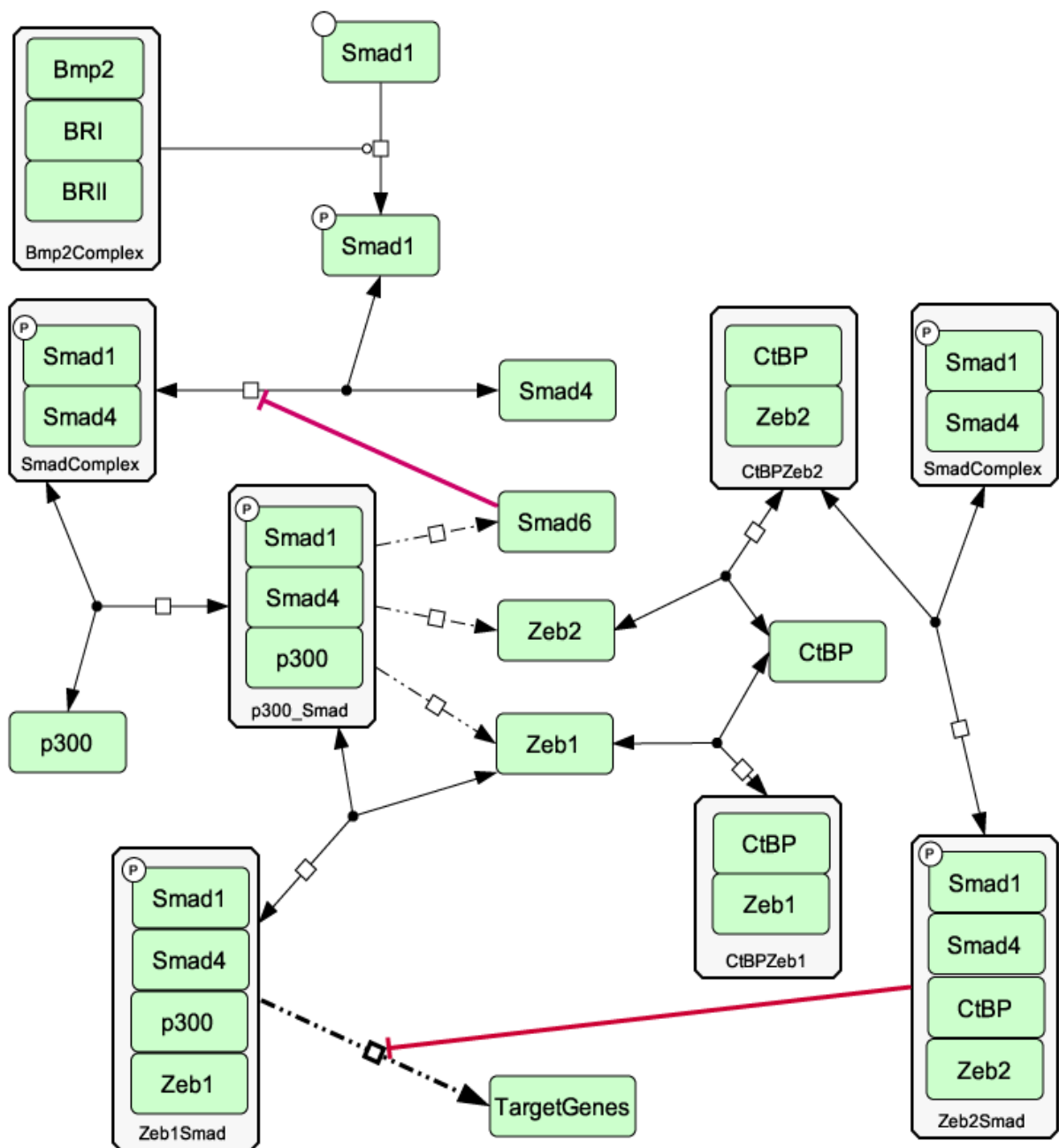

Supplement: Supplementary file 9 — Additional file 9: Figure S4. Computational simulation of Zeb1 and Zeb2 interaction in the context of BMP2-induced target gene expression. [file 40659_2023_417_MOESM9_ESM.pdf]
